# A Neutral Dy(II) Bis(amide): Synthesis, Magnetism, and a P_4_^2–^ Complex

**DOI:** 10.1021/acs.inorgchem.5c02908

**Published:** 2025-10-07

**Authors:** Rashmi Jena, Florian Benner, Richard J. Staples, Selvan Demir, Aaron L. Odom

**Affiliations:** Department of Chemistry, 3078 Michigan State University, 578 S. Shaw Ln, East Lansing, Michigan 48824, United States of America

## Abstract

Reaction of DyCl_3_ with KNHAr*, where Ar* = 2,6-Ar_2_C_6_H_3_ and Ar = 2,4,6-^i^Pr_3_C_6_H_2_, results in the formation of ClDy­(NHAr*)_2_, which can be reduced with KC_8_ in THF to room temperature stable dysprosium­(II) Dy­(NHAr*)_2_, where Ar rings of the NHAr* ligand chelate and bind to the metal center to give a black sandwich complex. The redox potential for the reversible Dy^2+/3+^-couple was found to be −1.094 ± 0.001 V with respect to FeCp_2_^+/0^ as 0.000 V. The complex was examined by CASSCF calculations, which suggest the non-Kramer’s ion has an 4f^9^5d^1^-configuration with little to no *s*-orbital participation. The reaction of Dy­(NHAr*)_2_ with CN^t^Bu results in assumed *tert*-butyl radical loss along with formation of the isocyanide CN–Dy­(NHAr*)_2_, which shows a strong absorption in the IR spectrum at 2052 cm^–1^ assigned to the C–N stretch. Reaction of P_4_ with Dy­(NHAr*)_2_ gave crystals of salt {Dy­(NHAr*)_2_}^+^{(P_4_)­Dy­(NHAr*)_2_}^−^, which shows both η^2^-P_4_^2–^ and η^4^-P_4_^2–^ ligands in the solid state.

## Graphical abstract

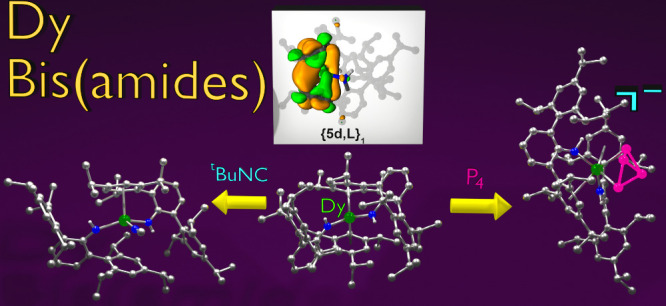

## Introduction

Lanthanides in oxidation states other than +3 offer a new and exciting area for the study of metal reactivity. Virtually, all of the lanthanides are now known in the +2 oxidation state,^1^ and we recently published a triarylamide, NHAr* (where Ar* = 2,6-Ar_2_C_6_H_3_ and Ar = 2,4,6-^i^Pr_3_C_6_H_2_), a framework that seems to reliably give stable *f*-block compounds in this lower oxidation state by coordination of pendant arenes,^2,3^ a strategy used by others in the community.^4–15^

Here, we discuss the synthesis and properties of the dysprosium­(II) complex Dy­(NHAr*)_2_ and its reactivity. Previous reports of Dy­(II) have largely focused on cyclopentadienyl ligands, but stable complexes with other ancillaries are known as well.^16–25^ In general, the focus seems to have been on the properties of the molecular Dy­(II) complexes once prepared, but we explore a couple of reactions here with *tert*-butylisocyanide, which makes an unusual CN–Dy­(NHAr*)_2_ isocyanide complex, and with P_4_, which makes {(P_4_)­Dy­(NHAr*)_2_}^−^ as what appears to be a mixture of η^2^-P_4_^2–^ and η^4^-P_4_^2–^ in a single disordered crystal.

## Results and Discussion

The H_2_NAr* preligand was prepared using the literature procedure.^26,27^ Deprotonation of H_2_NAr* with KCH_2_SiMe_3_ ^28^ gives the unstable potassium salt KNHAr*, which undergoes slow disproportionation to K_2_NAr* and H_2_NAr*, so it should be used on generation.^3^ The reaction of DyCl_3_ with 2 equiv of KNHAr* in diethyl ether gives ClDy­(NHAr*)_2_ (**1**) in 58% yield. The synthesis of this compound, and related {Dy­(NHAr*)_2_}^+^, along with their magnetic properties has been previously reported.^29^

### Synthesis and Properties of Dy­(NHAr*)_2_

The treatment of ClDy­(NHAr*)_2_ (**1**)^29^ with KC_8_ in THF results in a color change from the lemon yellow of **1** to black (Figure 1). Crystals from *n*-hexane examined by X-ray diffraction provide a sandwich structure for Dy­(II) bis­(amide) **2**. The average Dy–N distance in the Dy­(III) **1** is 2.247(3) Å,^29^ slightly shorter than in Dy­(II) **2** of 2.275(2) Å, as would be expected for the difference in oxidation state. However, the Dy–Ar­(centroid) distance in **2** is 2.4607(9) Å, significantly shorter than the Dy–Ar­(centroid) distance in Dy­(III) **1** ^29^ at 2.541(2) Å. This effect has been observed in U­(II)^2^ and Y­(II)^3^ complexes as well and is attributed to stronger M–Ar back-bonding in the lower oxidation-state metal centers.^2,3^ DFT calculations show a HOMO, LUMO, and unpaired spin density that are spread throughout the Ar–Dy–Ar axis. (See the SI for details.)

**Figure 1:**
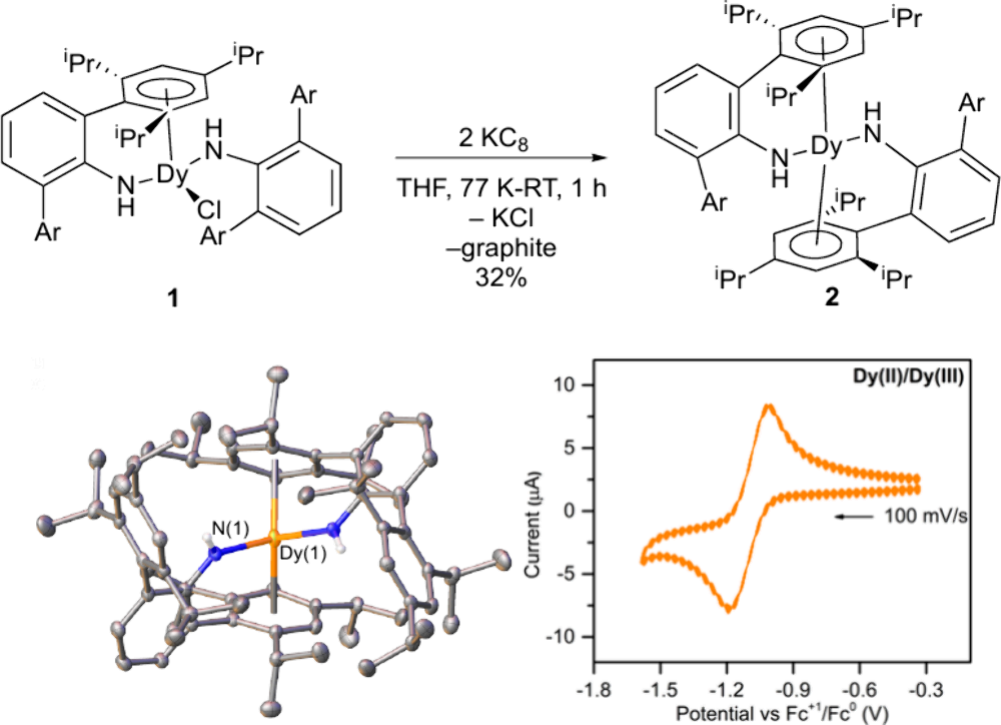
Synthesis of Dy­(NHAr*)_2_ (**2**). The structure is from single-crystal X-ray diffraction. See the SI for more details. Ellipsoids at the 30% probability level. A molecule of (disordered) THF is not shown. The molecule rests on a rotation axis, and only one of the ligands is unique by symmetry. Selected distances (Å) and angles (°): Dy(1)–N(1) = 2.275(2), Dy(1)–Ar­(centroid) = 2.4607(9), N(1)–Dy(1)–N(1)′ = 102.1(1), Ar­(centroid)–Dy–Ar′(centroid) = 134.40(7). Cyclic voltammogram in Et_2_O with 100 mM {NBu_4_}­{B­(Ar_F_)_4_}, where Ar_F_ = 3,5,-(CF_3_)_2_C_6_H_3_, with a glassy carbon working electrode and Pt wire pseudoreference and counter electrode.

The Ar­(centroid)–M–Ar­(centroid) angle in **2**, 134.40(7)°, is virtually identical to that in the related yttrium­(II) complex, 133.87(1)°.^3^

Complex **2** was examined by cyclic voltammetry under an argon atmosphere in diethyl ether. The experiment used 100 mM {NBu_4_}­{B­(Ar_F_)_4_}, where Ar_F_ = 3,5,-(CF_3_)_2_C_6_H_3_. The redox potential for the reversible Dy^2+/3+^-couple was found to be −1.094 ± 0.001 V with respect to FeCp_2_^+/0^ as 0.000 V. The scan rate for the plot shown in Figure 1 was 100 mV/s. However, the system seemed quite stable and was reversible down to 10 mV/s.

The Ln^2+/3+^ couple for this neutral Dy­(II) complex can be compared with the neutral Ln­(C_5_^i^Pr_5_)_2_^2+/3+^ complexes (Ln = Y, La, Ce, Pr, Nd, Gd, Ho, Er, Tm, Lu, Sm, Eu, Yb) prepared by Long, Harvey, and co-workers. Voltammograms were reported for Sm, Eu, Tm, and Yb, with the others undergoing decomposition under the experimental conditions. The stable Ln­(C_5_^i^Pr_5_)_2_^2+/3+^ complexes displayed *E*_1/2_ values from −1.92 (Sm) to −0.29 V (Eu), listed as quasi-reversible or irreversible in 1,2-difluorobenzene with 100 mV/s scan rates.^30^

The Dy­(II) complex **2** appears to be stable indefinitely at −35 °C in solution and for days at room temperature but, nevertheless, is quite reactive.

The magnetic properties of **2** were probed in the solid state on crushed crystalline samples via SQUID magnetometry. DC magnetic susceptibility measurements were carried out on **2** from 2 to 300 K under applied magnetic fields of 0.1, 0.5, and 1.0 T (Figure 2, Figures S17 and S18). At 0.1 T and 300 K, the χ_M_ *T* value for **2** is 14.86 cm^3^ K/mol, which gradually decreases until 10 K as the temperature is lowered, below which the decline is more prominent resulting in a χ_M_ *T* value of 6.22 cm^3^ K/mol at 2 K. Compared to the previously reported magnetic data of ClDy­(NHAr*)_2_ (**1**) bearing a Dy­(III) ion,^29^ the room temperature χ_M_ *T* value of **2** is considerably larger as is expected for the population of Dy-based 5d- or ligand-based π* orbitals.

**Figure 2:**
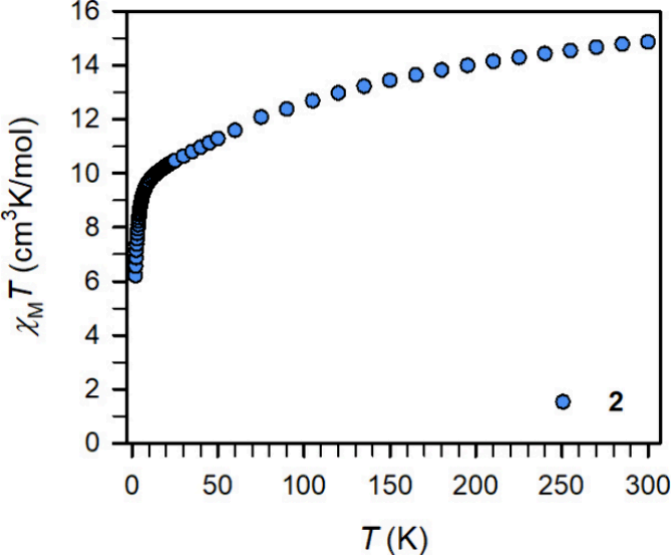
Temperature dependence of the χ_M_ *T* product for polycrystalline samples of **2** under a 1000 Oe applied dc field.

A comparison of the experimental room temperature χ_M_ *T* value for **2** of 14.86 cm^3^ K/mol to the expected value requires taking into consideration the conceivable electronic configurations. For a 4f^10^ Dy­(II) ion, the predicted value is 14.07 cm^3^ K/mol, which is much lower than the experimentally determined value for **2**.^21,23,30,31^ By contrast, the Ln­(II) ions best described with a 4f^*n*^5d^1^ configuration adhere to a L-S coupling scheme affording an expected value of 17.01 cm^3^ K/mol for a Dy­(II) ion.^21,30,31^ Reflecting on the result for **2**, the lower experimental χ_M_ *T* value hints at a devia from L-S coupling. An alternative interpretation for the magnitude of the room temperature χ_M_ *T* value is that it may arise from the strength of d-f coupling. If the d-f coupling is weak, a value of 14.55 cm^3^ K/mol is expected, whereas a strong d-f coupling would afford a value of 17.02 cm^3^ K/mol.^23^ Thus, a value of 14.86 cm^3^ K/mol is in line with weak d-f coupling and d-ligand coupling. Contributions from the 6s are expected to be negligible (see the *ab initio* section). Future advanced spectroscopic studies may provide insight onto such intricate electronic structure of a Ln­(II) ion as present in **2.**

As the amide ligands in **2** provide an axial ligand field to the metal center,^29,32–35^ ac magnetic susceptibility measurements were performed with a tiny oscillating ac field. Under zero applied dc field and even at the lowest temperatures, no definite, fully resolved out-of-phase (χ_M_″) signals were detected which may be attributed to the non-Kramers ion nature of Dy­(II). Variable-field ac magnetic measurements did not yield out-of-phase peaks either.

Field-dependent magnetization measurements (*M* vs *H*) on **2** were carried out between 0 and 7 T and 2 and 10 K (Figure S22). As expected from the ac magnetic results, the *M* vs *H* curves lack characteristics of magnetic blocking at low temperatures. At 2 K and at low fields, the magnetization increases to a value of 3.71 μ_B_ at 1.2 T, followed by a slower rise to a maximum value of 5.52 μ_B_ at 7 T. These maximum field μ_eff_ values are in excellent agreement with other divalent Dy complexes, which exhibit ∼5.5 μ_B_.^21,23^

The reduced magnetization curves (*M* vs *H*/*T*) reveal nonsuperimposable curves, suggesting substantial magnetic anisotropy (Figure 3). Thus, the lack of slow magnetic relaxation may be attributed to the non-Kramers ion nature of Dy­(II) with a nondegenerate ground state, rather than a lack of magnetic anisotropy.

**Figure 3:**
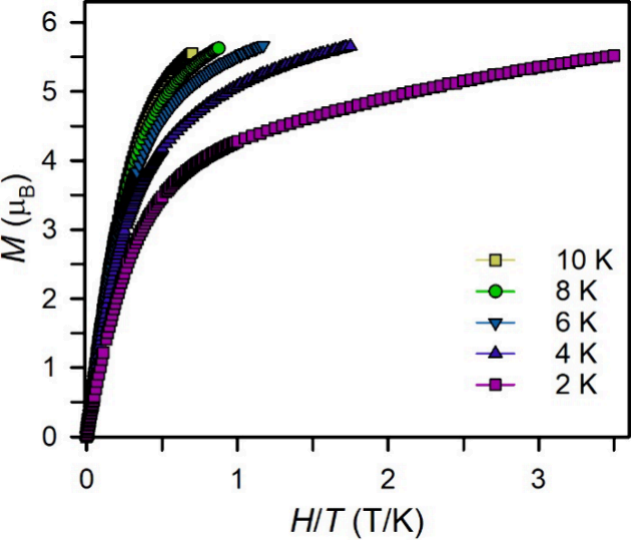
Reduced magnetization data for a polycrystalline sample of **2**, collected from 2 to 10 K, between 0 and 7 T.

A final check for single-molecule magnet behavior was conducted through the collection of magnetic hysteresis data at 1.8 K (Figure S20). The obtained hysteresis curve is fully closed, suggestive of rapid tunneling on the time scale of the variable-field measurement.

The curious reactivity of Dy­(NHAr*)_2_ (**2**) toward white phosphorus (vide infra) sparked our interest to investigate its electronic structure in greater detail. Especially when compared to the “classical” divalent lanthanide ions samarium, europium, thulium, and ytterbium, which exhibit 4f^*n*+1^ electronic configurations,^36^ the other lanthanides and yttrium may exhibit 4f^n^5d^1^ configurations.^23,37^ Since **2** exhibits a readily accessible reduction process at about −1.1 V (Figure 1 and Figures S15 and S16), considerably less negative compared to the recently reported, far more reducing Cp′_3_Dy (−2.96 V) (where Cp′ = C_5_H_4_SiMe_3_), we were particularly interested in uncovering the location of the experimentally injected electron through chemical reduction using theoretical methods.^38^

The stable Dy­(III) ion is multiconfigurational in nature due to its near-degenerate, fractionally occupied valence 4f orbitals. This necessitates the use of theoretical methods that can account for the multiconfigurational character such as the complete active space self-consistent field (CASSCF) method. For a trivalent Dy ion, the choice of an active space is rather straightforward since the 4f electrons are deeply buried beneath the 5d and 6s orbitals. Hence, the electrons can be safely assumed to reside within this shell. For the divalent Dy ion, however, this is not the case. Here, as alluded above, the chemically introduced additional electron can potentially populate several different shells, where 4f, 5d, 6s, 6p, or even ligand orbitals are conceivable, the latter depending on the ligand’s reduction potential.

Consequently, the choice of the active space requires careful investigation ahead of electronic structure analysis. Therefore, we conducted a set of spin–orbit-free CASSCF calculations using the ORCA 5.0.4 program suite scanning various Dy orbitals for their required presence in the active space (see Tables S1–S5).^39,40^ First, an active space comprising the seven 4f orbitals and 10 electrons with *S* = 2 (10,7) was chosen for reference and averaged over 35 roots. In a separate calculation, the 5d orbitals were added to the atomic 4f orbitals, resulting in an active space with *S* = 3 and 12 orbitals (10,12). Comparing the single point energies, the (10,12) wave function is clearly energetically favorable by 4.76 eV, rendering the 4f^*n*+1^ electronic structure for **2** unlikely (Tables S3 and S4). After orbital optimization of the (10,12) calculation, the five atomic 5d natural orbitals separated into two distinct groups: type 1 orbitals are strongly mixed with the π-orbitals of the capping phenyl rings, giving rise to two hybrid orbitals with ∼18% d-character and 0.51 electron occupancy plus a second ∼6% d-character/0.49 e^–^ occupancy orbital (Figures 4 and Figure S25). The other three 5d orbitals essentially retained their atomic character with occupancies close to zero. Such multiconfigurational electronic structures were observed for other organometallic complexes featuring divalent lanthanide ions such as {Ln­(Cp^iPr5^)_2_}^−^ (Ln = Tb, Dy).^41^ By contrast, test calculations on **2** including 5d and 6s atomic orbitals confirmed the strongly hybridized character of the fractionally occupied Dy 5d orbitals, while the 6s orbital could not be retained in the active space. This is in accordance with the negligible 6s contributions observed initially for both hybrid orbitals across all calculations (Tables S1 and S2), but starkly contrasts other reported divalent lanthanide complexes such as {Ln­(Piso)_2_} (Ln = Tb, Dy; Piso = {(NDipp)_2_C^t^Bu}, Dipp = 2,6-^i^Pr_2_C_6_H_3_), where almost 1/1 ratios for 5d/6s hybrids were detected.^23^

**Figure 4:**
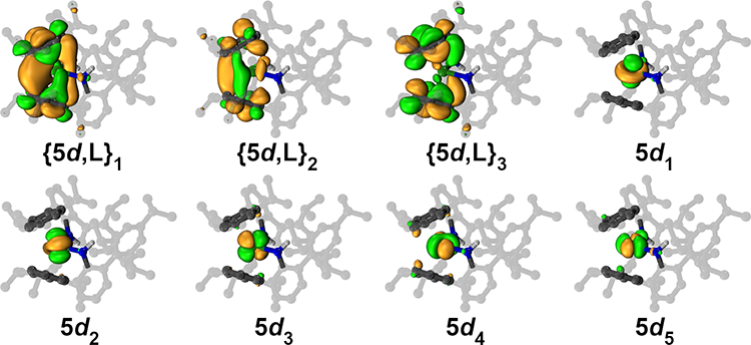
Natural orbitals obtained from the (10,15) CASSCF calculation on Dy­(NHAr*)_2_ (**2**). The state-averaged occupation numbers are {5d,L}_1_ (0.514), {5d,L}_2_ (0.314), {5d,L}_3_ (0.172), 5d_1_ (0.002), 5d_2_ (0.002), 5d_3_ (0.002), 5d_4_ (0.002), and 5d_5_ (0.002). The corresponding 4f orbitals with occupation numbers of 1.284 were omitted for clarity. An isovalue of 0.03 was used for plotting in the VMD visualization software.^42^

In the final calculation, we included ligand-based orbitals into the active space since the two hybrid orbitals obtained throughout are very similar to the π* orbitals derived from the calculation of the hypothetical metal-free {(NHAr*)_2_}^2–^ fragment. The (10,15) calculation considered the five 5d and three lowest-lying ligand π* orbitals resulting in three hybrid orbitals with 0.51:0.31:0.17 e^–^ occupations, while the five 5d orbitals retained their atomic character with ∼0 e^–^ occupation. The hybrid orbitals gained up to 18% Dy character, highlighting the intricate electronic structure of **2**. In sum, the calculations suggest that the electronic structure of **2** is best described as an intermediate between (A) a “true” Dy­(II) ion and (B) a Dy­(III) ion coordinated to an organic radical.

Lastly, the spin ground state of **2** was probed by employing the orbitals obtained from the (10,15) calculation while simultaneously accounting for a potential inverted spin in the 5d shell as opposed to the 4f shell (Table S5). The state-averaged calculation over 35 *S* = 3 and 35 *S* = 2 states hints at the sextet ground state to be more favorable by ∼1037 cm^–1^ over the quartet ground state with spin–orbit coupling unconsidered.

### Reaction of Dy­(NHAr*)_2_ with CN-*t*-Bu

The treatment of Dy­(NHAr*)_2_ (**2**) with CN-*t*-Bu in *n*-hexane results in a rapid color change of the solution from black to pale yellow (Figure 5). X-ray-quality, yellow crystals were obtained from *n*-hexane solution at low temperature. Similar to other *XM*(NHAr*)_2_ complexes recently prepared (where *X* = Cl, NC, and I; and *M* = U, Y, and Dy),^2,3,29^ **3** has only one arene interacting with the metal center. The Dy–N3­(amide) distance, where the amide does not have a chelating arene, is somewhat shorter at 2.218(2) Å than the Dy–N2­(amide) distance with chelation at 2.237(2) Å, presumably due to slightly less favorable Dy–N overlap when the arene chelates. The Dy–Ar­(centroid) distance is 2.5090(2) Å, longer than Dy­(II) **2** but somewhat shorter than chloride **1.**

**Figure 5:**
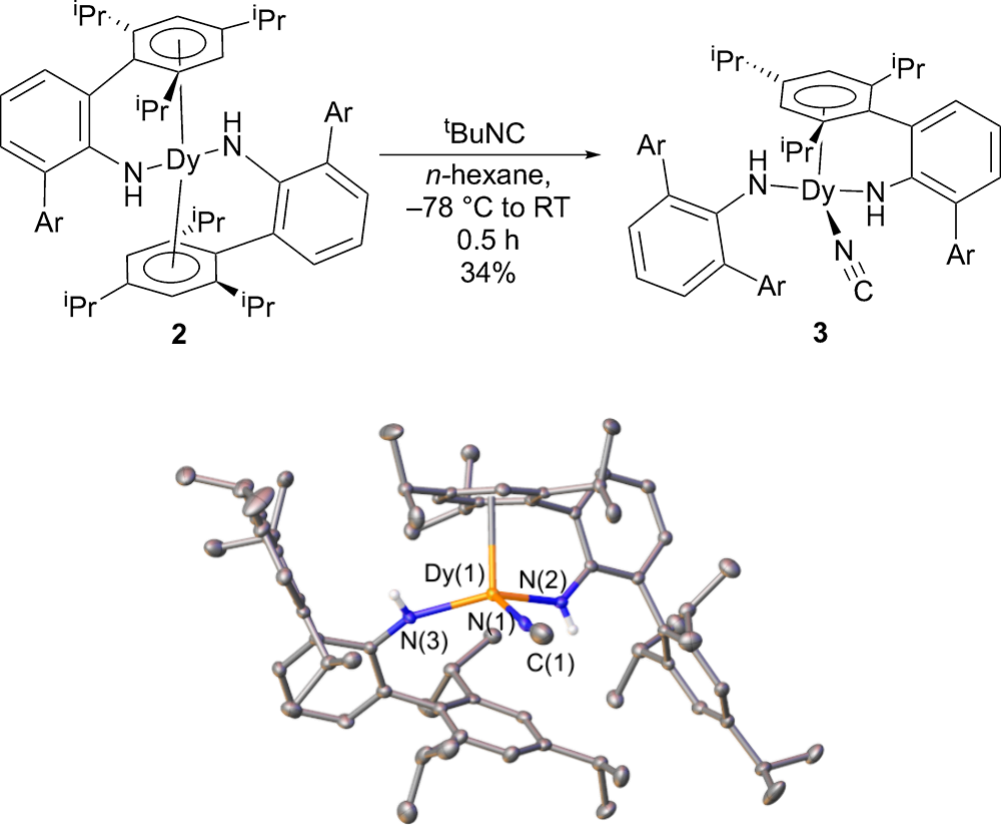
Synthesis of CN–Dy­(NHAr*)_2_ (**3**). The structure is obtained from single-crystal X-ray diffraction. See the SI for more details. *n*-Hexane in the lattice is not shown. Selected distances (Å) and angles (°): Dy(1)–N(3) = 2.218(2), Dy(1)–N(2) = 2.237(2), Dy(1)–N(1) = 2.372(2), Dy–Ar­(centroid) = 2.5178(2), N–Dy–*N* = 102.1(1), and C(1)–N(1)–Dy(1) = 174.6(2).

We assign **3** to the isocyanide structure, similar to the yttrium analog,^3^ based on several factors. (1) The structure from X-ray diffraction has better refinement statistics when solved with the nitrogen bound to Dy. For the isocyanide CN–Dy­(NHAr*)_2_ structure, *R*_1_ = 0.0364 and *wR*_2_ = 0.0864, for *I* > 2σ­(*I*). When the diatomic is reversed to the more typical cyanide NC–Dy­(NHAr*)_2_, *R*_1_ = 0.0378 and *wR*_2_ = 0.0914. (2) The infrared stretching frequencies for **3**, 2052 cm^–1^, and CN–Y­(NHAr*)_2_, 2053 cm^–1^, are virtually identical. There is strong spectroscopic evidence from ^13^C–^89^Y NMR coupling that the yttrium analog is an isocyanide, and the IR stretching frequencies are the same, suggesting the same structure.^3^ In addition, based on literature data for low back-bonding metal centers, the stretching frequency for isocyanides is ∼2050 cm^–1^, about 100 cm^–1^ lower than in cyanides, which is due to differences in lone pair bond weakening when the carbon and nitrogen lone pairs are bonded.^3^ (3) DFT calculations (TPSSh/def2-SVP) were carried out on the isocyanide complex. There is reasonably good agreement between the gas phase calculation and the solid-state structure. For example, the experimental/calculated distances (Å) are Dy(1)–N(2) = 2.218(2)/2.2756, Dy(1)–N(3) = 2.237(2)/2.285, Dy(1)–N(1) = 2.372(2)/2.332, and Dy–Ar­(centroid) = 2.5178(2)/2.570. The isocyanide CN–Dy­(NHAr*)_2_ (**3**) was found to be 2.5 kcal/mol lower in energy than that of the NC–Dy­(NHAr*)_2_ complex. The frequency calculation for ν_CN_ in **3** was remarkably close at 2132 cm^–1^ (cf., experimental value of 2052 cm^–1^); expectedly, the calculated ν_CN_ in cyanide NC–Dy­(NHAr*)_2_ was higher at 2218 cm^–1^.

Compound **3** appears to be the first reported example of a dysprosium isocyanide complex. A few *f*-block systems previously have been reported with metal isocyanides.^43–48^ In addition, a magnesium system has been found to undergo isomerization between cyanide and isocyanide.^49^ Since both the HOMO and LUMO of CN^–^ reside largely on the carbon, when such structures are observed, they seem to be under charge control rather than molecular orbital control. As a result, metal isocyanides may be restricted to highly polar, low back-bonding systems.^3^

### Reaction of Dy­(NHAr*)_2_ with P_4_

Dysprosium­(II) complex **2** reacts with P_4_ in benzene at room temperature, with a color change from black to orange occurring over about an hour. The reaction requires two equiv of **2**, as electron transfer occurs to generate the salt {Dy­(NHAr*)_2_}^+^ {(P_4_)­Dy­(NHAr*)_2_}^−^ (**4**) (Figure 6).

**Figure 6:**
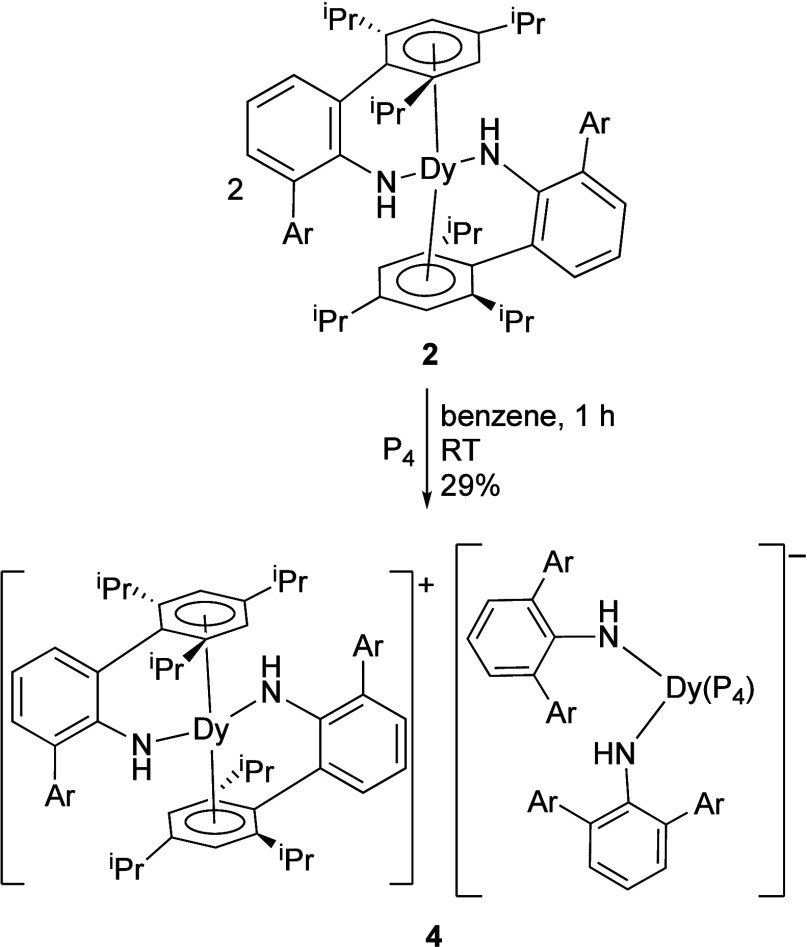
Synthesis of {Dy­(NHAr*)_2_}^+^{(P_4_)­Dy­(NHAr*)_2_}^−^ (**4**).

Multiple batches of orange crystals from different reactions were grown separately from either diethyl ether or toluene for examination by single-crystal X-ray diffraction. The experiment on the crystal grown in ether was done at 100 K. Two structures were obtained on a crystal from toluene at 100 and 200 K. (The crystal lost solvent and decomposed at 300 K.) The best data was obtained from diethyl ether where the crystals were kept as cold as possible from the mother liquor to the instrument.

The cation {Dy­(NHAr*)_2_}^+^ in **4** has been previously observed as the B­(Ar_F_)_4_^–^ salt, where Ar_F_ = 3,5-(CF_3_)_2_C_6_H_3_, and the metrical parameters are similar to the structure from X-ray diffraction with the alternative counterion.^29^

The anion {(P_4_)­Dy­(NHAr*)_2_}^−^ is quite unusual. It seems to be ∼4-coordinate in the solid state (depending on how the P_4_ unit is counted, *vide infra*) with only bonds to phosphorus and amide nitrogens. In the minor isomer with approximately equal Dy–P distances, the closest arene approach is 3.049 Å for one of the ipso-carbons of the arenes, while the other Dy–C distances range out to 3.447 Å. The Dy–C distance to the bound arene in **3** for example is 2.807–2.937 Å. If there is an arene-Dy interaction in this isomer of **4**, then it is relatively weak. The closest Dy–C distance in the major isomer of **4** is 3.198 Å, and it is assumed that there is no arene interaction.

Complexes of P_4_ with transition metals have been extensively examined with fascinating results.^50,51^ Often, the phosphorus will give a *cyclo*-P_4_^2–^ unit,^52–54^ which is isolobal with *cyclo*-C_4_H_4_^2–^. Also known are some η^1^-P_4_ complexes, where the unit is a phosphine-like donor.^55–61^ Two different kinds of η^2^-P_4_ complexes are in the literature: a neutral form where all the bonds of the tetrahedral P_4_ are retained^62–64^ and a dianionic form where one of the P–P bonds in P_4_ is broken^65–73^ with those phosphorus atoms now bonded to a metal(s). The η^4^-P_4_^2–^ (*cyclo*-P_4_^2–^) and η^2^-P_4_^2–^ are then the two known isomeric forms for the ligand in this oxidation state.

In the anion of **4** (Figure 7) structures best described as η^2^-P_4_^2–^and η^4^-P_4_^2–^ (*cyclo*-P_4_^2–^) derivatives are observed in the solid-state disordered structure. In the structure containing diethyl ether within the lattice, the η^2^-P_4_^2–^: η^4^-P_4_^2–^ ratio was found to be 70:30. From the two structures of crystals grown from toluene and examined at different temperatures, the model suggested essentially identical η^2^-P_4_^2–^: η^4^-P_4_^2–^ ratios of 78:22 (100 K) and 79:21 (200 K).

**Figure 7:**
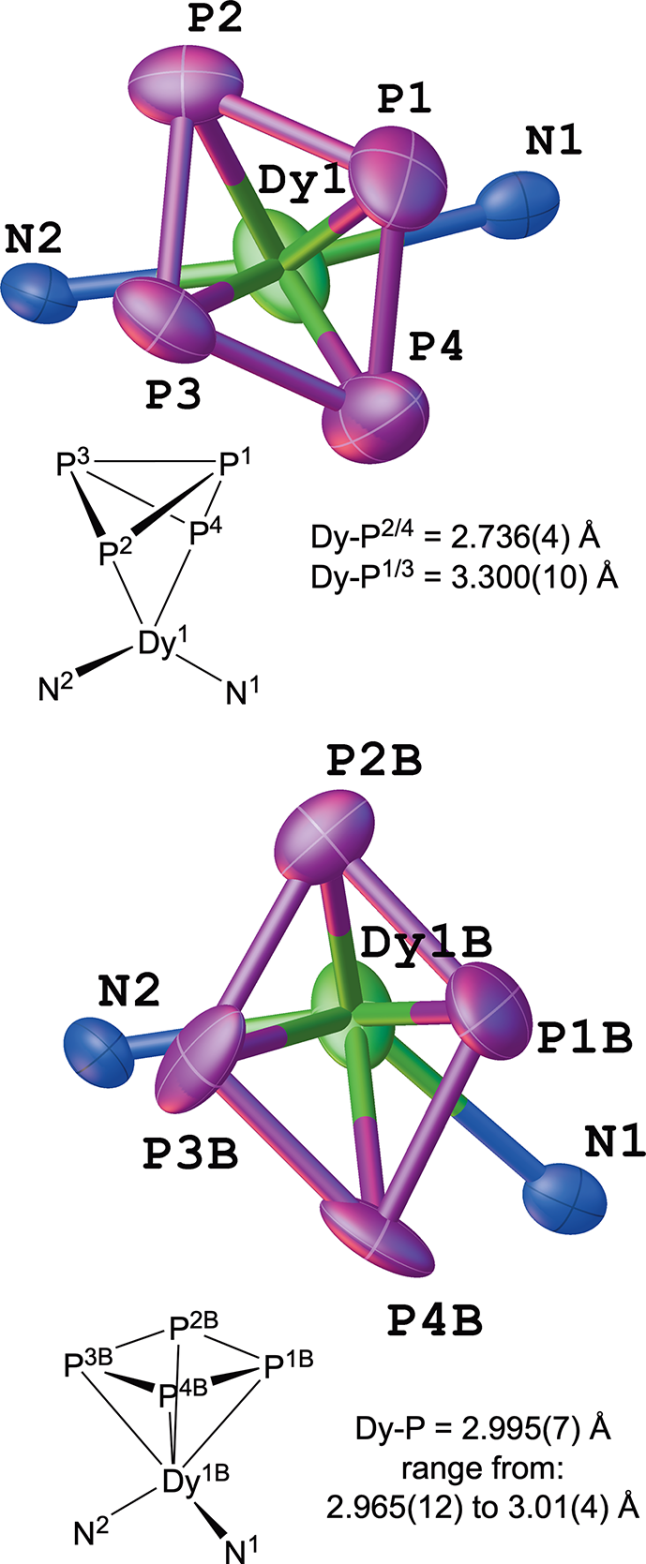
Structure of {Dy­(NHAr*)_2_}^+^{(P_4_)­Dy­(NHAr*)_2_}^−^ (**4**) from single-crystal X-ray diffraction emphasizing the resolved disorder from the structure containing diethyl ether and taken at 100 K. See the SI for more details. The P_4_ fragment is disordered between η^4^-P_4_^2–^ (30%) and η^2^-P_4_^2–^ (70%).

The isolation of two different geometric isomers in a complex is unusual but not unprecedented. In one extreme case, Raymond and co-workers found that the Ni­(CN)_5_^3–^ ion could be either trigonal bipyramidal or square pyramidal.^74,75^ In this case, there is a pocket formed by the large NHAr* ligands that can be occupied by either P_4_^2–^ geometric isomer (Figure 7), leading to a disordered structure.

Complex **4** is the first observation of an *f*-block η^2^-P_4_^2–^ complex, which is the major isomer in the structure, but there are quite a few cases where η^2^-P_4_^2–^ units have been observed with transition metals.^65–73,76^ These η^2^-P_4_^2–^ complexes are most often found bridging multiple metal centers, but there are a few where there is a single metal chelated by the P_4_ unit, as found in **4**. For example, Scherer and co-workers reported that Cp″M­(CO)_2_, where *M* = Zr/Hf and Cp″ = 1,3-(^t^Bu)_2_C_5_H_3_, reacts with P_4_ to give MCp″_2_(η^2^-P_4_) complexes in quantitative yield.^50^ Figueroa and co-workers described the reaction of Fe­(CNAr)_2_(CO)_2_(N_2_) with P_4_, which gives Fe­(CNAr)_2_(CO)_2_(η^2^-P_4_) in 86% yield.^76^

There are only a few *f*-element complexes with P_4_^2–^ ligands, all of them containing *cyclo*-P_4_ units between multiple metal centers.^77–79^ Again, the first structurally characterized *f*-block case of a η^2^-P_4_^2–^complex seems to be **4**. In this case, both are observed. What is the energy difference between these two isomeric P_4_^2–^ ligands, and when is one preferred over the other?

As shown in Figure 7, the major isomer containing η^2^-P_4_^2–^ has very different Dy–P distances, as would be expected. The two phosphorus atoms bound to Dy are on average 2.74 Å from the metal, while the unbound atoms have a distance of 3.3 Å. The P–P bond shown in the top structure of the η^2^-P_4_^2–^ compound is 2.297(9) Å; this is slightly longer than the P–P bonds around the edge of the ring, averaging 2.161(12) Å. Interestingly, this is quite the opposite of the situation in Figueroa and co-workers Fe­(η^2^-P_4_)­(CNAr)_2_(CO)_2_ complex, where the edge P–P bonds average 2.222 Å and the diagonal P–P bond is 2.188 Å.^76^ The P–P–P angles subtended at the phosphorus atoms bound to Dy are quite acute at 64.3°, while other P–P–P angles (excluding the diagonal bond) of the rhombohedral P_4_ unit are 96.0°.

Examination of the minor isomer is hindered by higher esds than those in the major isomer but gives a clearly different picture. The minor isomer assigned as a cyclo-η^4^-P_4_^2–^ has essentially equal Dy–P bond distances of 3.00(4), 2.97(1), 3.01(1), and 3.01(4) Å. Geometrically, the P_4_ unit in the minor isomer exhibits features suggestive of being between the η^2^ and η^4^ limiting forms being discussed. The P–P–P angles have a short and long pair pattern similar to that of the η^2^-P_4_ form at 104.93°, 70.15°, 108.34°, and 72.40°, but the system is approximately planar with these angles summing to 355.8°. The P–P bond distances around the ring are 2.01(2), 2.18(1), 2.18(1), and 2.17(1) Å, similar to the distances in the other isomer, but the closest diagonal distance is 2.44(2) Å, significantly longer than the diagonal P–P bond in the other isomer. It is difficult to know if the distorted angles are due to an electronic feature, the relatively low quality of the minor isomer structure, or steric constraints in the local environment, but it appears to be most consistent with a *cyclo*-η^4^-P_4_^2–^ assignment.

We examined this computationally in a couple of ways (Figure 8). First, we calculated the energy difference between isolated P_4_^2–^ isomers in the gas phase. The aromatic P_4_^2–^ cyclic compound has a calculated enthalpy (M06/aug-cc-pVTZ) that is lower by 10 kcal/mol in the absence of a Lewis acid. Next, we looked at the effect of having a poorly back-bonding Lewis acid on the enthalpy and found that the η^2^-structure was preferred by 17 kcal/mol when it can chelate to a single metal center. However, if the cationic charge is more evenly distributed on the two sides of the P_4_^2–^ plane, like in Na_2_P_4_, then the planar form becomes more favorable.

**Figure 8:**
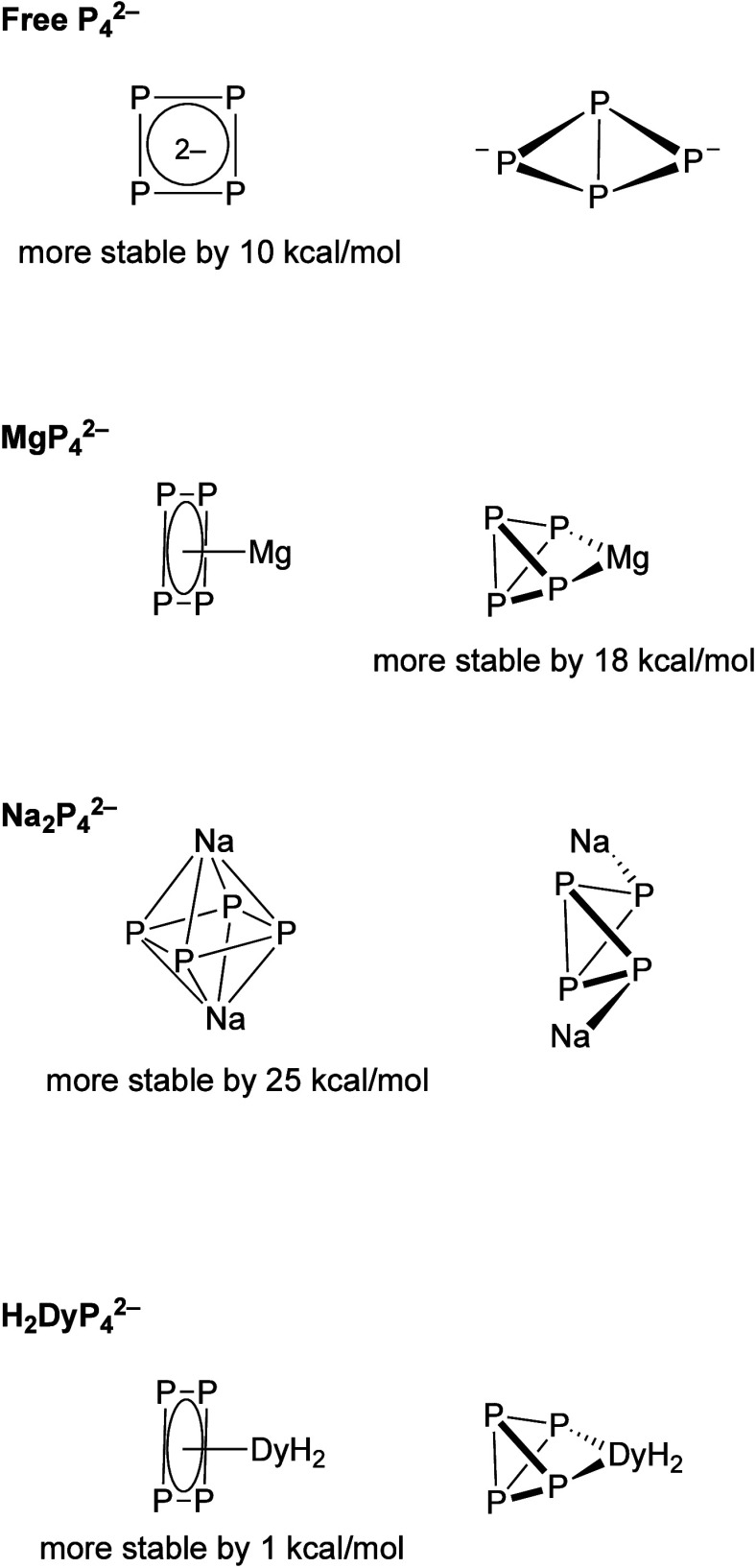
Calculations are for the gas phase using M06/aug-cc-PVTZ. The energies are for the differences in enthalpies between the two structural minima shown.

Calculations (TPSSh/def2-SVP) on a model complex, P_4_DyH_2_, gave that the difference in enthalpy between the η^2^- and η^4^-forms was 0.9 kcal/mol (Figure 8). We were also able to find the transition state converting η^2^-P_4_^2–^ and η^4^-P_4_^2–^ ligands in the model system, and the barrier was a substantial 30 kcal/mol. The calculations were redone with P_4_Dy­(NHPh)_2_ as the model, and the difference in enthalpy between the η^4^- and η^2^-forms was a similar 1.5 kcal/mol with a 30 kcal/mol barrier.

Based on calculations using the entire structure of the anion in **4** (TPSSh/def2-SVP), we found that the η^2^-P_4_^2–^ and η^4^-P_4_^2–^ structures were about 1 kcal/mol different in free energy, very similar to the P_4_DyH_2_ and P_4_Dy­(NHPh)_2_ models. Unfortunately, we were unable to locate the transition state converting between the two structures, but as mentioned, two different model structures gave 30 kcal/mol as the barrier (vide supra).

The calculations on the full anion of **4**, {(η^2^-P_4_)­Dy­(NHAr*)_2_}^−^, suggest that the η^2^-P_4_^2–^ has a more localized HOMO indicative of anionic charge on 2 of the 4 phosphines that act as donors toward the *f*-block metal. Conversely, the η^4^-P_4_^2–^ containing anion has a much more delocalized HOMO across all of the phosphorus atoms, the metal, and ancillary ligands. (See the SI for more information on the calculations.)

## Conclusions

Here, we successfully isolated Dy­(NHAr*)_2_ (**2**), which is isostructural to the yttrium and uranium analogs.^2,3^ The Vis-NIR spectroscopy, room-temperature magnetic susceptibility value (χ_M_T), and DFT calculations suggest that the ground-state electronic configuration for **2** is 4f^9^5d^1^. SQUID measurements and *ab initio* calculations support a 4f^9^5d^1^ electronic configuration for **2**, which is comparable to some other Ln­(II) complexes.^21,23,31,37^ However, unlike such examples, **2** features negligible 5d-6s mixing in favor of 5d-ligand mixing giving rise to negligible magnetic coupling between 4f and 5d shells. Furthermore, the magnetism and computational suggestion of d-orbital occupation are consistent with the broad, Laporte-allowed 5d-4f electronic transition in **2** observed at 755 nm with ε = 780 M^–1^ cm^–1^. The data suggest some Dy­(III)/organic radical character mixing into the “Dy­(II)” complex **2** due to back-bonding into the aromatic systems of the ligands.

The highly reactive metal radical **2** reacts with CN-*t*-Bu to give a rare metal isocyanide CN–Dy­(NHAr*)_2_ (**3**) complex after the loss of the *tert*-butyl radical. DFT calculations suggest that the metal isocyanide is more stable than cyanide. This reaction shows charge-controlled reactivity like the yttrium analog. This appears to be the first structurally characterized example of a dysprosium isocyanide complex.

Treatment of **2** with elemental P_4_ resulted in the formation of anionic {(P_4_)­Dy­(NHAr*)_2_}^−^ (**4**) with Dy­(NHAr*)_2_^+^ as a counterion. The doubly reduced P_4_ showed a mixture of isomers on the metal center with 70% η^2^-P_4_^2–^ and 30% cyclic η^4^-P_4_^2–^. Calculations suggest that the η^2^-P_4_^2–^ and η^4^-P_4_^2–^ are around ∼1 kcal/mol different in energy and likely are both found in solutions of the complex. A few cyclic η^4^-P_4_^2–^ complexes have been reported previously,^77–79^ but the η^2^-P_4_^2–^ isomer appears to be the first structurally characterized example of such a complex in the *f*-block.

## Experimental Section

### General Considerations

All manipulations were done under purified nitrogen atmosphere in either a glovebox or using Schlenk techniques. Hexamethyldisiloxane and *n*-hexane were dried over CaH_2_ and, after sparging with dinitrogen, were passed through alumina. Diethyl ether was purified by passing through alumina columns to remove water after being sparged with dry nitrogen to remove oxygen. 2,6-dichloroiodobenzene was purchased from Oakwood and, after freeze–pump–thawing three times to remove dissolved gases, was transferred into the glovebox. *tert*-Butyl isocyanide and trimethylacetonitrile were purchased from Sigma-Aldrich and, after freeze–pump–thawing three times to remove dissolved gases, were passed through activated alumina before use. Complex Dy­(NHAr*)_2_Cl (**1**) was synthesized using the literature procedure.^29^

**Caution!** Metal amides, white phosphorus, and KC_8_ are highly reactive, potentially pyrophoric compounds that can react violently with water and oxygen.

### Synthesis of Metal Complexes

#### Synthesis of Dy­(NHAr*)_2_(**2**)

A 20 mL scintillation vial was charged with crystals of Dy­(NHAr*)_2_Cl (**1**) (272.8 mg, 0.22 mmol, 1 equiv), THF (5 mL), and a magnetic stir bar. The vial was placed in a liquid nitrogen-cooled cold well until the solution froze. Once frozen, the vial was removed from the cold well to a magnetic stir plate. When the solution had thawed enough to stir, a suspension of KC_8_ (61.9 mg, 0.45 mmol, 2 equiv) in THF (2 mL) was added. The solution rapidly turned color from yellow to black. The reaction was stirred for 1 h at room temperature. The volatiles were removed *in vacuo*, and the remaining residue was dissolved in *n*-hexane and filtered twice through Celite using a pipette filter. Black-colored X-ray-quality single crystals were grown by chilling a concentrated *n*-hexane solution of **2** in a −35 °C freezer overnight (85.7 mg, 32.4% yield). The complex was stable for months at −35 °C. The crystals contain one THF molecule per metal complex. C_72_H_100_N_2_Dy·C_4_H_8_O: C, 74.30; H, 8.87; N, 2.28. Found: C, 73.99; H, 9.45; N, 2.14. λ_max_ (nm): 755 (at higher concentrations), 297 (at lower concentrations).

#### Synthesis of Dy­(NHAr*)_2_NC (**3**)

A 20 mL scintillation vial was charged with **2** (12.7 mg, 0.01 mmol, 1 equiv), *n*-hexane (1 mL), and a magnetic stir bar. A separate 20 mL scintillation vial was loaded with *tert*-butylisonitrile (5.1 mg, 0.01 mmol, 1 equiv) and *n*-hexane (1 mL). Both solutions were cooled in a dry ice/acetone cold well for 20 min, and then, the CN^*t*^Bu solution was added dropwise into the solution of **2**. The solution color changed rapidly from black to pale yellow. The reaction mixture was allowed to stir for 30 min. The solvent was removed *in vacuo*. The resulting yellow solid was extracted with *n*-hexane and filtered through Celite. The solvent was removed *in vacuo*. A concentrated solution of the product in *n*-hexane resulted in the formation of yellow-colored X-ray quality single crystals overnight at −35 °C in the freezer (4.2 mg, 0.003 mmol, 34% yield). Anal. Calcd for C_73_H_100_N_3_Dy: C, 74.22; H, 8.53; N, 3.56. Found: C, 73.95; H, 8.72; N, 3.47. λ_max_ (nm): 1439, 1402, 1316, 1249, 1192 (at higher concentrations), 297 (at lower concentrations). ν_NC_ = 2052 cm^–1^.

#### Synthesis of [Dy­(NHAr*)_2_]­[Dy­(NHAr*)_2_P_4_] (**4**)

A 20 mL scintillation vial was charged with **2** (31.5 mg, 0.025 mmol, 1 equiv), benzene (2 mL), and a stir bar. A separate 20 mL scintillation vial was loaded with white phosphorus (1.5 mg, 0.012 mmol, 0.5 equiv) and benzene (1 mL). To the solution of **2**, white phosphorus was added. The reaction color changed from black to orange after 1 h of stirring at room temperature. The solvent was removed *in vacuo*. The orange solid was dissolved in diethyl ether and filtered through Celite. A concentrated solution of the product in diethyl ether resulted in the formation of orange-colored X-ray-quality single crystals after 2–3 days at −35 °C in the freezer (10.8 mg, 0.008 mmol, 29% yield). We further recrystallized our sample using toluene layered with *n*-hexane, which gave slightly better diffracting crystals after 3–4 days at −35 °C. Anal. Calcd for C_144_H_200_N_4_Dy_2_P_4_: C, 70.96; H, 8.21; N, 2.29. Found: C, 70.86; H, 8.69; N, 2.23. λ_max_ (nm): 1192, 1247, 1399, 1445, 297.

## Supplementary Material



## Notes

Additional characterization data and computational details (PDF)
