# Supplementary material for: A Neutral Dy(II) Bis(amide): Synthesis, Magnetism, and a P4 2– Complex
Source: Inorg Chem. 2025 Oct 7;64(41):20643–51. doi: 10.1021/acs.inorgchem.5c02908 (PMC12541703; doi:10.1021/acs.inorgchem.5c02908)
Supplement: Supplementary file 1 [file ic5c02908_si_001.pdf]

Supporting Information

A Neutral Dy(II) Bis(amide): Synthesis, Magnetism, and a P_4^{2-} Complex

Rashmi Jena, Florian Benner, Richard J. Staples, Selvan Demir, and Aaron L. Odom**

Michigan State University, Department of Chemistry, 578 S. Shaw Ln, East Lansing, MI, 48824, United States.

*Correspondence to: odoma@msu.edu (A. L. O) or sdemir@chemistry.msu.edu (S.D.)

Table of Contents

UV-Vis-Near IR Spectra 6

Figure S1. Vis-NIR spectrum of $Dy(NHAr^*)_2$ (**2**) obtained from 1 mM solution in ether. The values of molar absorptivity are approximated from one measurement. 6

Figure S2. UV-vis spectrum of $Dy(NHAr^*)_2$ (**2**) obtained from serial dilutions. 7

Figure S3. Concentration vs absorbance plot to calculate molar extinction coefficient at 297 nm for $Dy(NHAr^*)_2$ (**2**) in diethyl ether. The slope of the line provides ε = 19307 M^{-1} cm^{-1}. 7

Figure S4. Vis-NIR spectrum of $Dy(NHAr^*)_2NC$ (**3**) obtained from 7.4 mM solution in ether. The values of molar absorptivity are approximated from one measurement. 8

Figure S5. UV-vis spectrum of $Dy(NHAr^*)_2NC$(**3**) obtained from serial dilutions. 8

Figure S6. Concentration vs absorbance plot to calculate molar extinction coefficient at 297 nm for $Dy(NHAr^*)_2NC$ (**3**) in diethyl ether. The slope of the line provides ε = 7518 M^{-1} cm^{-1}. 9

Figure S7. Vis-NIR spectrum of $[Dy(NHAr^*)_2][(P_4)Dy(NHAr^*)_2]$ (**4**) obtained from 1.9 mM solution in ether. The values of molar absorptivity are approximated from one measurement...... 9

Figure S8. UV-vis spectrum of $[Dy(NHAr^*)_2][Dy(NHAr^*)_2P_4]$ (**4**) obtained from serial dilutions. 10

Figure S9. Concentration vs absorbance plot to calculate molar extinction coefficient at 297 nm for $[Dy(NHAr^*)_2][Dy(NHAr^*)_2P_4]$ (**4**) in diethyl ether. The slope of the line provides ε = 40425 M^{-1} cm^{-1}.. 10

Figure S10. Concentration vs absorbance plot to calculate molar extinction coefficient at 400 nm for $[Dy(NHAr^*)_2][Dy(NHAr^*)_2P_4]$ (**4**) in diethyl ether. The slope of the line provides ε = 18687 M^{-1} cm^{-1}.. 11

FT-IR Spectroscopy 11

Figure S11. FT-IR spectrum for complex $Dy(NHAr^*)_2$ (**2**) in the solid state. 12

Figure S12. FT-IR spectrum for complex $Dy(NHAr^*)_2NC$ (**3**) in the solid state....................... 12

Figure S13. Zoomed in FT-IR spectrum for complex $Dy(NHAr^*)_2NC$ (**3**) (~5 μM) in *n*-hexane. 13

Figure S14. FT-IR spectrum for complex $[Dy(NHAr^*)_2][Dy(NHAr^*)_2P_4]$ **4** in the solid state. . 13

Cyclic Voltammetry 14

Figure S15. Cyclic voltammogram of 2 in Et_2O (1.2 mM) with $[NBu_4][B(3,5\text{-}(CF_3)_2C_6H_3)_4]$ (100 mM) as supporting electrolyte and $Fc^{+/0}$ as 0 V. This voltammogram was scanned in the negative direction with 100 mV/s scan rate. 14

Figure S16. Overlay plot for scan rates in diethyl ether with $[NBu_4]^+[B(3,5\text{-}(CF_3)_2C_6H_3)_4]^-$ (100 mM) as supporting electrolyte and $Fc^{+/0}$ as 0 V.. 15

Magnetic Measurements 15

Figure S17. Temperature dependence of the $\chi_M T$ product for a polycrystalline sample of **2** under a 5000 Oe applied dc field. 16

Figure S18. Temperature dependence of the $\chi_M T$ product for a polycrystalline sample of **2** under a 1.0 T applied dc field. 16

Figure S19. Variable-temperature *M*(*H*) data curves for **2** collected from 0 to 7 T. 17

Figure S20. Plot of magnetization (*M*) vs dc magnetic field (*H*) at an average sweep rate of 100 Oe/s for **2** at 1.8 K. 17

Ab initio calculation 18

Table S1. State-averaged occupation numbers of the optimized active orbitals obtained from different CASSCF calculations over 35 roots. The 4*f* orbital occupation numbers are in accordance with 9 electrons throughout. Since CASSCF natural orbitals are employed, the 4*f* and 5*d* orbitals are mixtures of different *f*/*d* orbitals, respectively, and thus, numbers were assigned rather than using a description of their atomic orbitals. Hybrid orbitals are indicated with {}. 19

Table S2. Composition of active space orbitals after orbital optimization for Dy 5*d*, 6*s*, and 6*p* contributions. 19

Table S3. Relative spin-free energies for different CASSCF calculations for 35 roots of the high spin state of [$(NHAr^*)_2Dy$] (**2**) $S = 3$ in cm^{-1}. 20

Table S4. Summary of single point energies obtained from different CASSCF calculations. 21

Table S5. Relative energies of the high spin $S = 3$ and low spin $S = 2$ states obtained from the final CASSCF (10,15) calculation in cm^{-1}. The energy of the first state $S = 3$ state is 1036.6 cm^{-1} lower than the first $S = 2$ state. 21

Single Crystal X-ray Diffraction 22

Figure S21. Asymmetric unit of $Dy(NHAr^*)_2$ (**2**) recrystallized from THF. Complex **2** crystallizes with one molecule of THF in the lattice. Ellipsoids at the 30% probability level. 23

Figure S22. Structure of $Dy(NHAr^*)_2NC$ (**3**) recrystallized from *n*-hexane. Half a molecule of *n*-hexane is in the lattice per molecule of **3**. Ellipsoids at the 30% probability level. 24

Table S6. Thermal parameters for carbon and nitrogen atoms in $Dy(NHAr^*)_2NC$ (**3**) vs $Dy(NHAr^*)_2CN$ (**3'**) crystal structure. 25

Table S7. Metric data from the crystal structures of $Dy(NHAr^*)_2$ (**2**) $Dy(NHAr^*)_2NC$ (**3**), 25

Structure Analysis of [$Dy(NHAr^*)_2$][$Dy(NHAr^*)_2P_4$] (4) 26

Figure S23. Structure of [$Dy(NHAr^*)_2$][$Dy(NHAr^*)_2P_4$] **4** recrystallized from diethyl ether. Three molecules of diethyl ether are in the lattice per molecule of **4**. Ellipsoids at the 30% probability level. 28

Figure S24. (top) Zoom-in for P_4^{2-} butterfly (left) and planar (right) form. (bottom) Structural metrics for **4** from single-crystal x-ray diffraction. 30

Table S8. Comparison of counter cation $[Dy(NHAr^*)_2]^+$ from structure **4** to $[Dy(NHAr^*)_2][BArF_{24}]$ structure........ 31

Table S9. Crystallographic data and structural refinement of $Dy(NHAr^*)_2Cl$ (**1**), $Dy(NHAr^*)_2$ (**2**), and $Dy(NHAr^*)_2NC$ (**4**)........ 31

DFT Calculations........ 32

Table S10. Structural comparisons between crystallographically obtained geometry of **2** and optimized geometry using DFT calculations. Shown here are some characteristic lengths (Å) and angles (˚). 33

Figure S25. HOMO (top, left), LUMO (top, right), and spin density map (bottom) of **2**. 34

Figure S26. Optimized structures of $Dy(NHAr^*)_2NC$ (**3**) (left) and $Dy(NHAr^*)_2CN$ (**3'**) (right). All hydrogens except N–H hydrogens are removed for clarity. 35

Table S11. Structural comparisons between crystallographically obtained geometry of $Dy(NHAr^*)_2NC$ (**3**) and optimized geometry using DFT calculations. Shown here are some characteristic bond lengths (Å) and angles (˚). 35

Figure S27. Optimized structures of butterfly-$[(NHAr^*)_2Dy–P_4]^-$ (left) and pseudo-planar $[(NHAr^*)_2Dy–P_4]^-$ (right). All hydrogens except N–H hydrogens are removed for clarity. 36

Table S12. Structural comparisons between crystallographically obtained geometry of butterfly- and pseudo planar-$[(NHAr^*)_2Dy–P_4]^-$, and optimized geometry using DFT calculations. Shown here are some characteristic bond lengths (Å) and angles (˚). 36

Figure S28. Frontier molecular orbitals for butterfly-$[(NHAr^*)_2Dy–P_4]^-$ structure........ 38

Figure S29. Frontier molecular orbitals for cyclo-$[(NHAr^*)_2Dy–P_4]^-$ structure........ 38

Table S13. Relative enthalpies (TPSSh/def2-SVP) of butterfly and cyclo-binding mode of P_4^{2-} unit with transition state. 39

Optimized Geometry Coordinates 39

$Dy(NHAr^*)_2$ (**2**) 39

Dy(NHAr*)NC (**3**)........ 45

Dy(NHAr*)$_2$CN (**3'**) 51

Butterfly-[(NHAr*)$_2$Dy(P$_4$)]$^-$ structure (**4**) 57

Planar-[(NHAr*)$_2$Dy(P$_4$)]$^-$ structure (**4**) 63

References 70

UV-Vis-Near IR Spectra

The electronic absorption spectra were recorded on a double-beam PerkinElmer 1050 spectrophotometer. The measurement was done in a 1 cm pathlength quartz cell at a ~1 mM concentration of complexes **2**, and **3**. Preparation of samples was performed in the N_2 glovebox using dry ether. The raw data were fitted with OriginPro 9.0 software to obtain accurate maxima assuming Gaussian peak shapes. Low concentrations of **2**, **3**, and **4** were monitored to observe charge transfer bands. UV-Vis spectra were collected using an Ocean Optics DH-mini UV-Vis spectrophotometer in an N_2 glovebox. A series of solutions were prepared with varying concentrations through serial dilutions to determine the molar extinction coefficients for charge transfer bands. All spectra were baseline-corrected for diethyl ether and collected at ambient temperature.

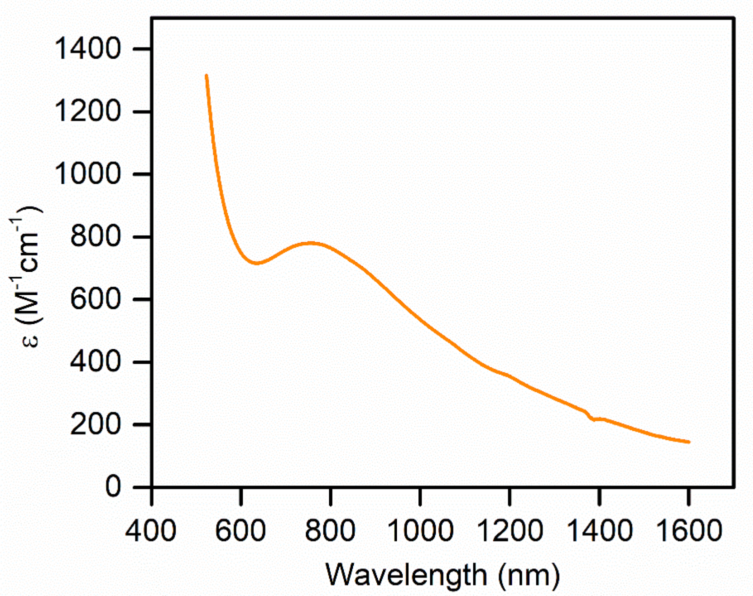

Figure S1. Vis-NIR spectrum of Dy(NHAr*)$_2$ (**2**) obtained from 1 mM solution in ether. The values of molar absorptivity are approximated from one measurement.

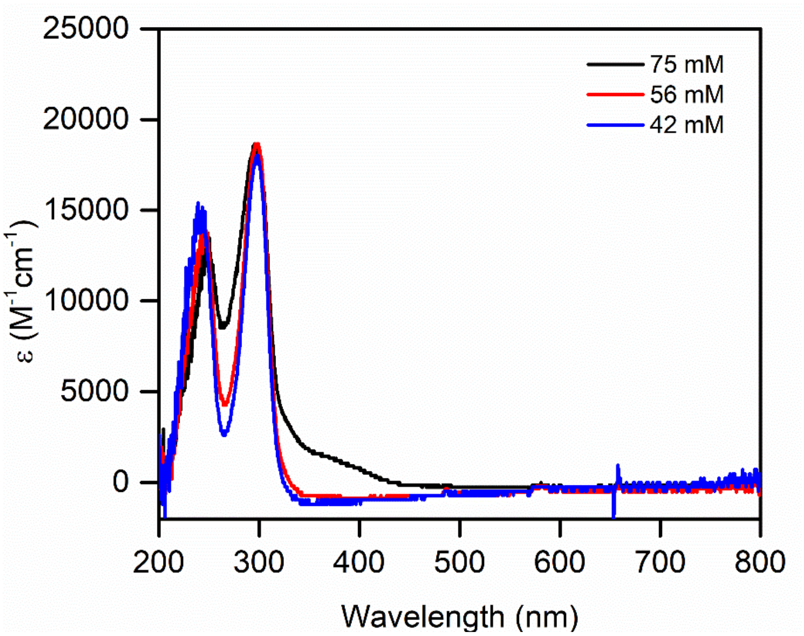

Figure S2. UV-vis spectrum of Dy(NHAr*)$_2$ (**2**) obtained from serial dilutions.

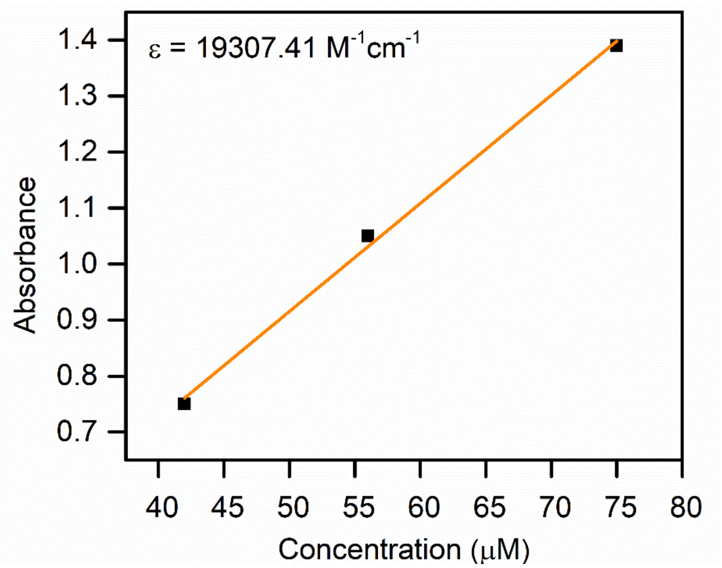

Figure S3. Concentration vs absorbance plot to calculate molar extinction coefficient at 297 nm for Dy(NHAr*)$_2$ (**2**) in diethyl ether. The slope of the line provides $\varepsilon = 19307\ M^{-1}\ cm^{-1}$.

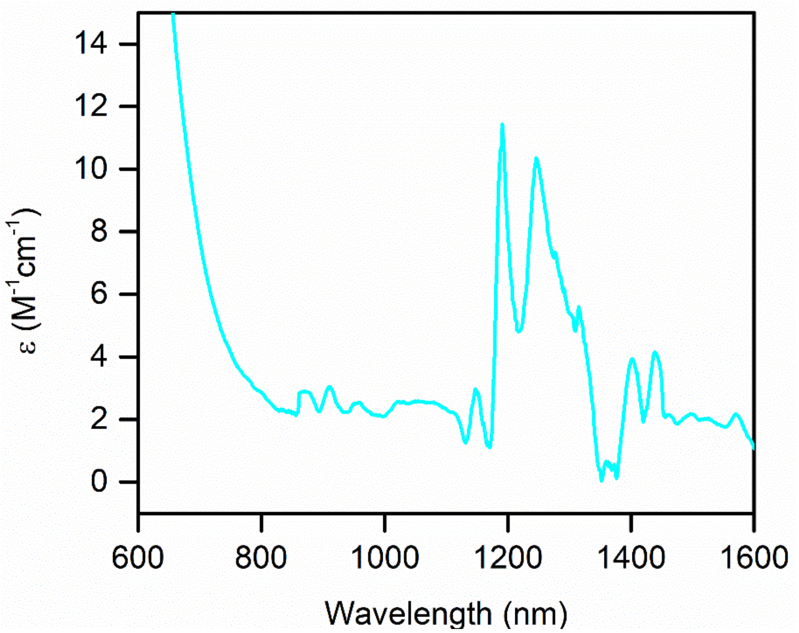

Figure S4. Vis-NIR spectrum of Dy(NHAr*)$_2$NC (**3**) obtained from 7.4 mM solution in ether. The values of molar absorptivity are approximated from one measurement.

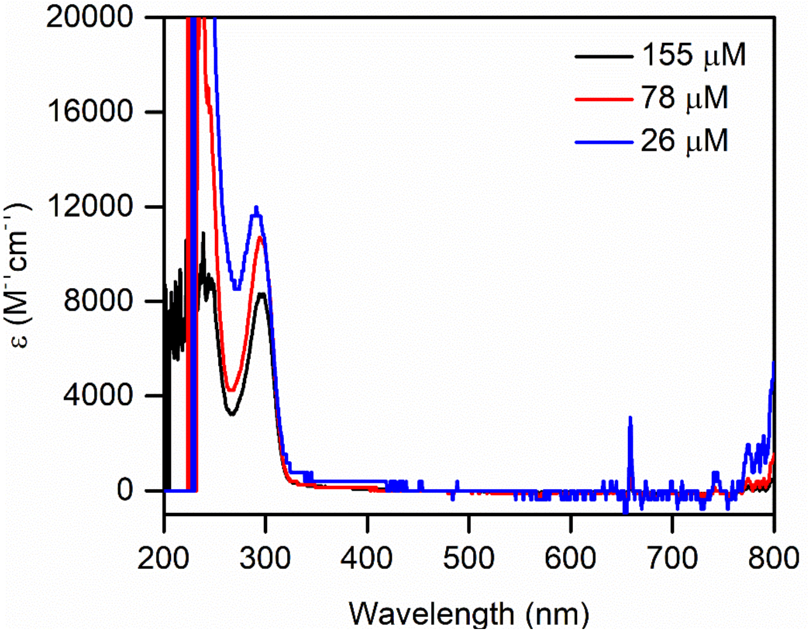

Figure S5. UV-vis spectrum of Dy(NHAr*)$_2$NC(**3**) obtained from serial dilutions.

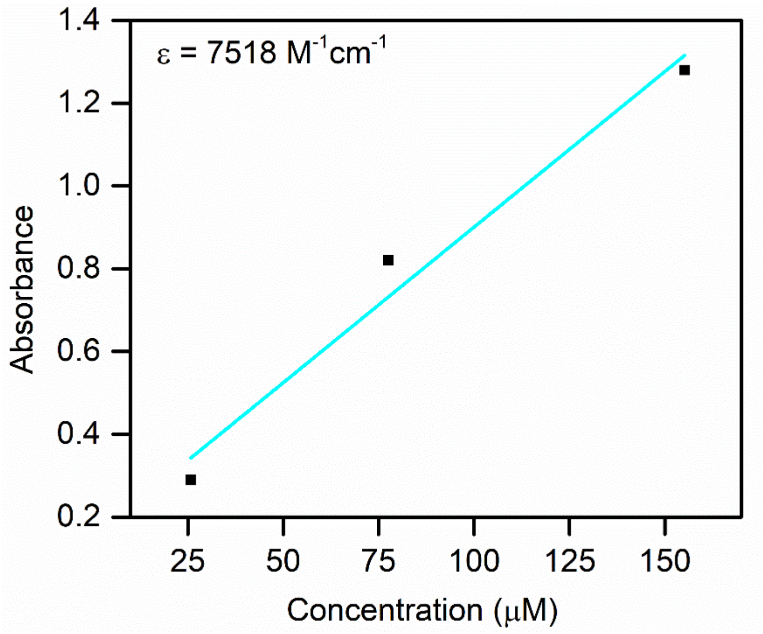

Figure S6. Concentration vs absorbance plot to calculate molar extinction coefficient at 297 nm for Dy(NHAr*)$_2$NC (**3**) in diethyl ether. The slope of the line provides ε = 7518 M^{-1} cm^{-1}.

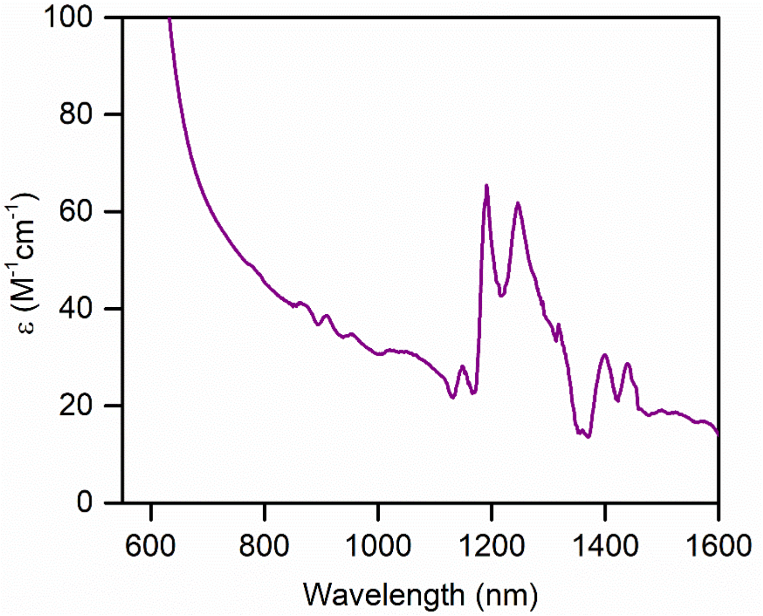

Figure S7. Vis-NIR spectrum of [Dy(NHAr*)$_2$][(P$_4$)Dy(NHAr*)$_2$] (**4**) obtained from 1.9 mM solution in ether. The values of molar absorptivity are approximated from one measurement.

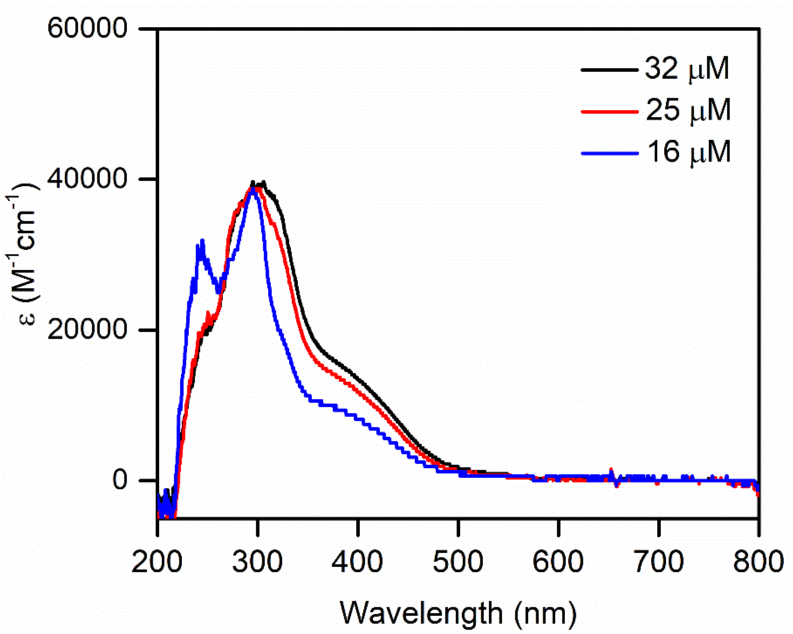

Figure S8. UV-vis spectrum of [Dy(NHAr*)$_2$][Dy(NHAr*)$_2$P$_4$] (**4**) obtained from serial dilutions.

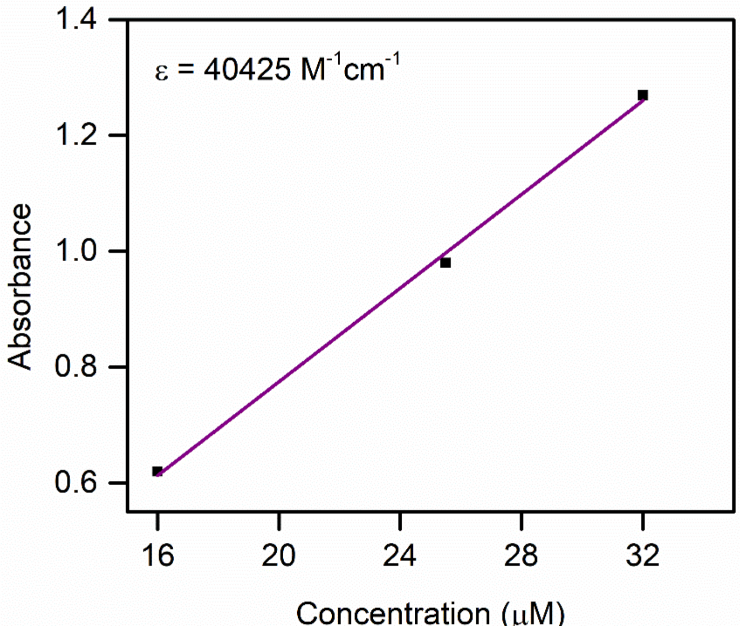

Figure S9. Concentration vs absorbance plot to calculate molar extinction coefficient at 297 nm for [Dy(NHAr*)$_2$][Dy(NHAr*)$_2$P$_4$] (**4**) in diethyl ether. The slope of the line provides ε = 40425 M^{-1} cm^{-1}.

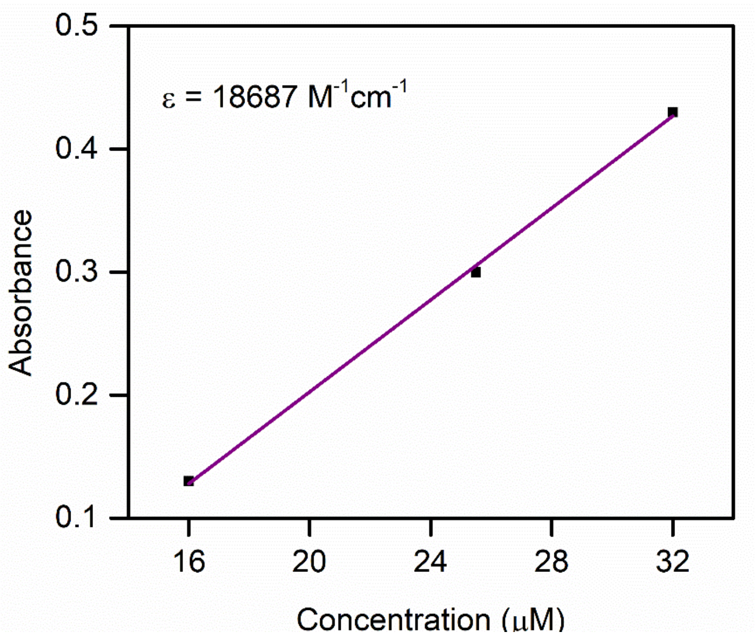

Figure S10. Concentration vs absorbance plot to calculate molar extinction coefficient at 400 nm for $[Dy(NHAr^*)_2][Dy(NHAr^*)_2P_4]$ (**4**) in diethyl ether. The slope of the line provides ε = 18687 M^{-1} cm^{-1}.

FT-IR Spectroscopy

IR Spectra were recorded using Varian 3100 FT-IR spectrometer with a spectral resolution of 2 cm^{-1} using a CaF_2 air-free cell. Sample preparation was done in the glove box under an N_2 atmosphere and sealed with Teflon caps before measurements. The spectrum was recorded in the solution phase using dry *n*-hexane as solvent. The IR transmittance spectra were collected at 298 K and the baseline was corrected. The ν_{NC} stretch for $Dy(NHAr^*)_2NC$ (**3**) is 2051.9 cm^{-1} lies in the range of previously reported value metal isocyanides.[2–5]

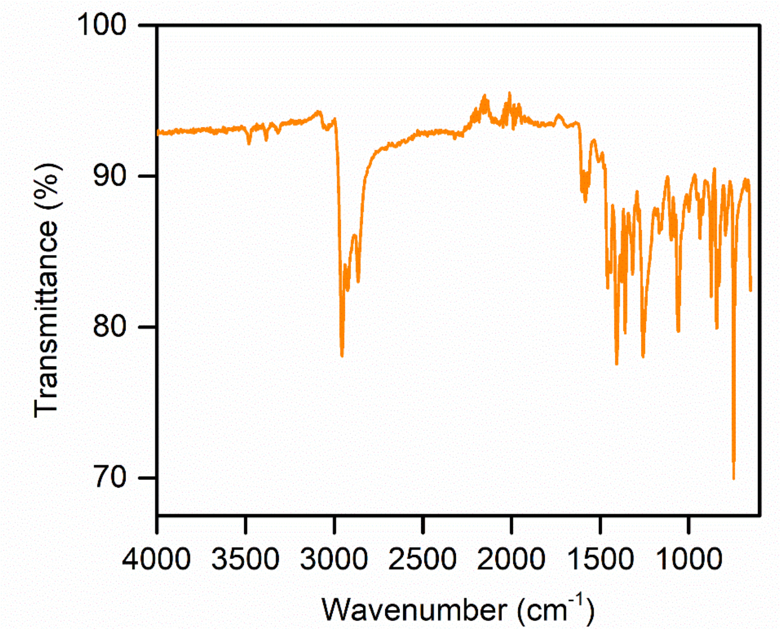

Figure S11. FT-IR spectrum for complex Dy(NHAr*)$_2$ (**2**) in the solid state.

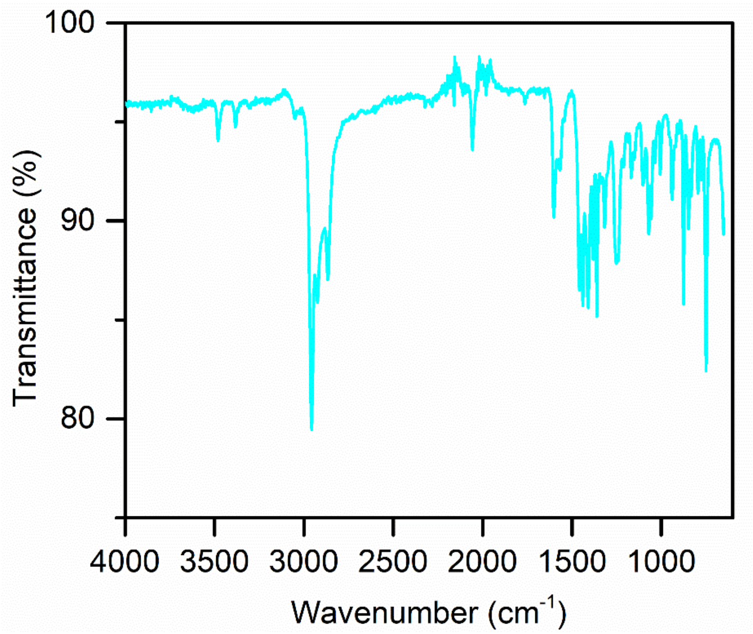

Figure S12. FT-IR spectrum for complex Dy(NHAr*)$_2$NC (**3**) in the solid state.

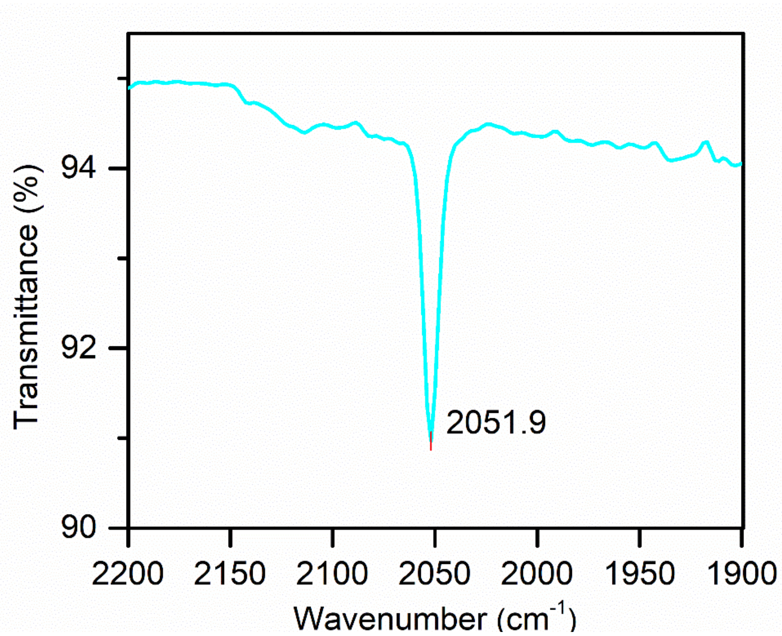

Figure S13. Zoomed in FT-IR spectrum for complex Dy(NHAr*)$_2$NC (**3**) (~5 μM) in *n*-hexane.

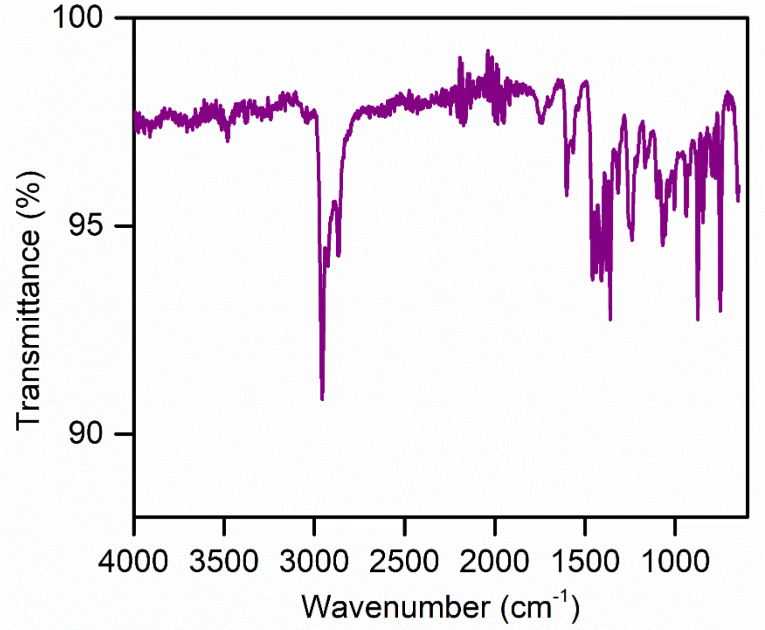

Figure S14. FT-IR spectrum for complex [Dy(NHAr*)$_2$][Dy(NHAr*)$_2$P$_4$] **4** in the solid state.

Cyclic Voltammetry

Cyclic voltammetry was recorded using a PGSTAT204 potentiostat from Metrohm with a glassy carbon working electrode, platinum wire pseudo-reference electrode, and platinum wire as a counterelectrode. All measurements were in the glovebox under argon atmosphere. Dy(NHAr*)$_2$ **2** (1.2 mM) was dissolved in diethyl ether with $[NBu_4]^+[B(3,5\text{-}(CF_3)_2C_6H_3)_4]^-$ (100 mM) as supporting electrolyte. Cyclic voltammetry of ferrocene was performed six times to get standard deviation in potential shifts. The reduction potential for Fc^+/Fc^0 redox couple was observed at 1.14 ± 0.4 V. All voltammograms were externally referenced to ferrocene with the same electrolyte concentration. All measurements were done in triplicate to get an average value of half-cell potential. The redox potential for Dy(II)/Dy(III) couple is 1.094 ± 0.001 V with respect to Fc^+/Fc^0 couple. The reversibility of this one-electron redox process was studied at different scan rates from 90 mV/s to 60 mV/s with 10 mV/s intervals.

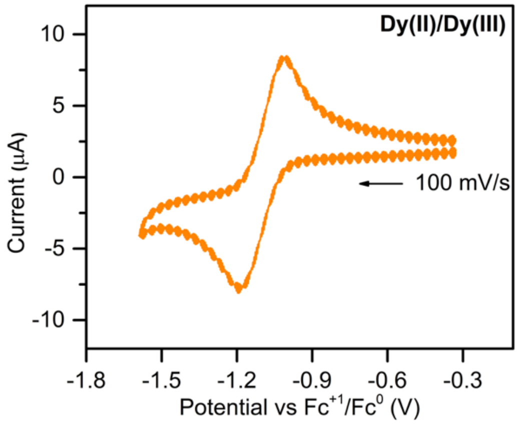

Figure S15. Cyclic voltammogram of 2 in Et_2O (1.2 mM) with $[NBu_4][B(3,5\text{-}(CF_3)_2C_6H_3)_4]$ (100 mM) as supporting electrolyte and $Fc^{+/0}$ as 0 V. This voltammogram was scanned in the negative direction with 100 mV/s scan rate.

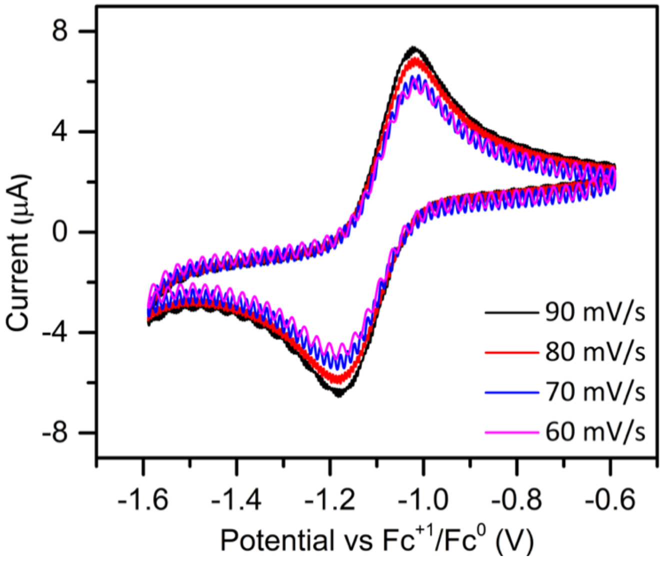

Figure S16. Overlay plot for scan rates in diethyl ether with $[NBu_4]^+[B(3,5\text{-}(CF_3)_2C_6H_3)_4]^-$ (100 mM) as supporting electrolyte and $Fc^{+/0}$ as 0 V.

Magnetic Measurements

Magnetic susceptibility measurements were performed on a Quantum Design MPMS3 Super-conducting Quantum Interference Device (SQUID) magnetometer. The magnetic sample of $Dy(NHAr^*)_2$, **2**, (where Ar* = $2,6\text{-}Ar_2C_6H_3$ and Ar = $2,4,6\text{-}{}^iPr_3C_6H_2$), was prepared by saturating and covering dried, crushed crystalline solids of **2** (17.7 mg, 1.531×10^{-5} mol) with molten eicosane (71.6 mg) at 40 °C to prevent crystallite torquing and to provide good thermal contact between the sample and the bath. The samples were sealed in an airtight container and transferred to the magnetometer. All data were corrected for diamagnetic contributions from the eicosane, and core diamagnetism was estimated using Pascal's constants.[6]

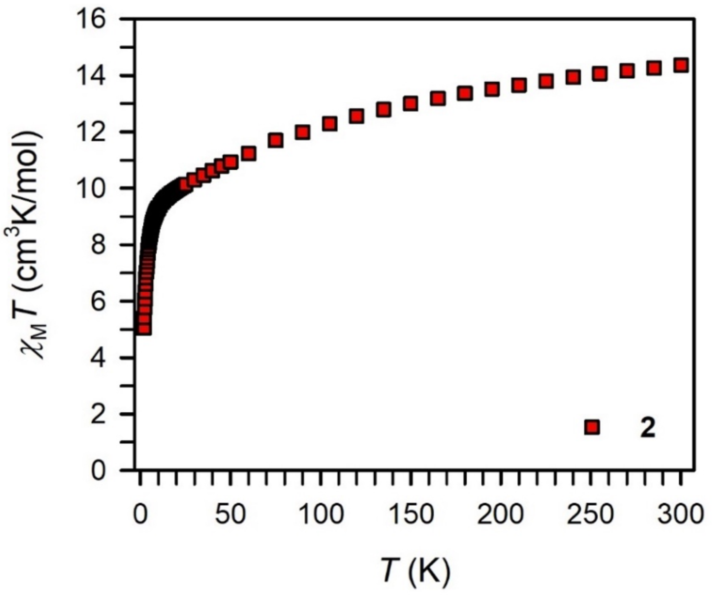

Figure S17. Temperature dependence of the $\chi_M T$ product for a polycrystalline sample of **2** under a 5000 Oe applied dc field.

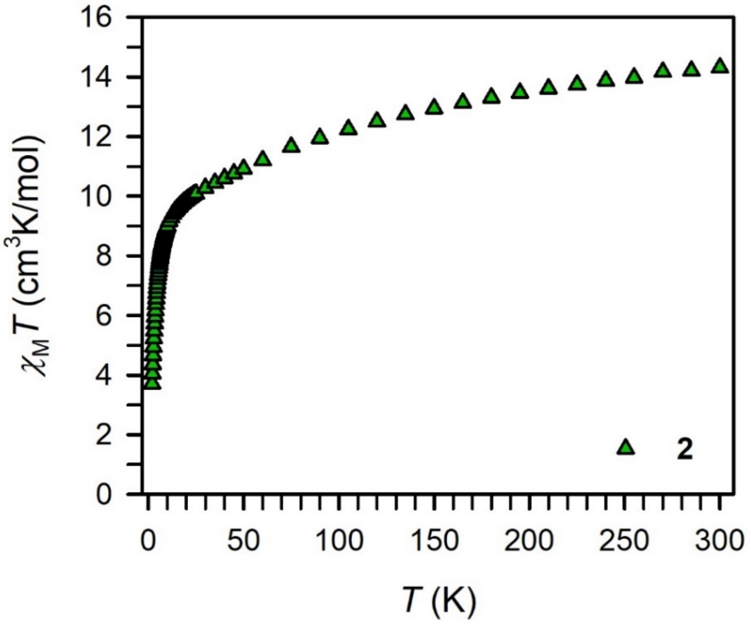

Figure S18. Temperature dependence of the $\chi_M T$ product for a polycrystalline sample of **2** under a 1.0 T applied dc field.

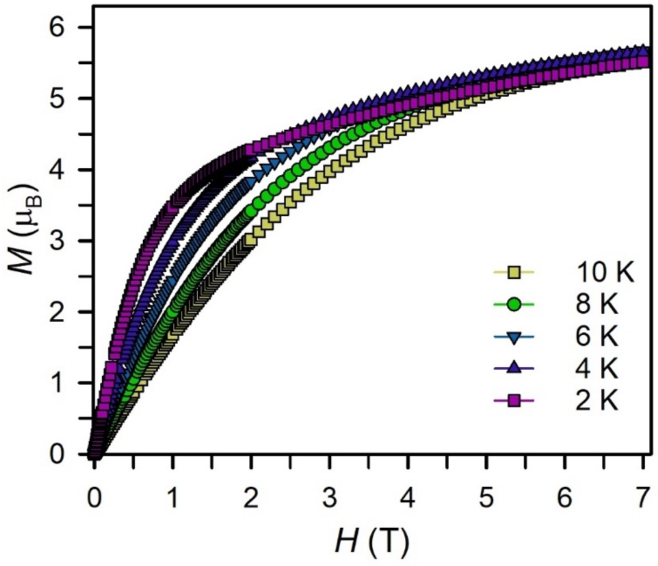

Figure S19. Variable-temperature *M*(*H*) data curves for **2** collected from 0 to 7 T.

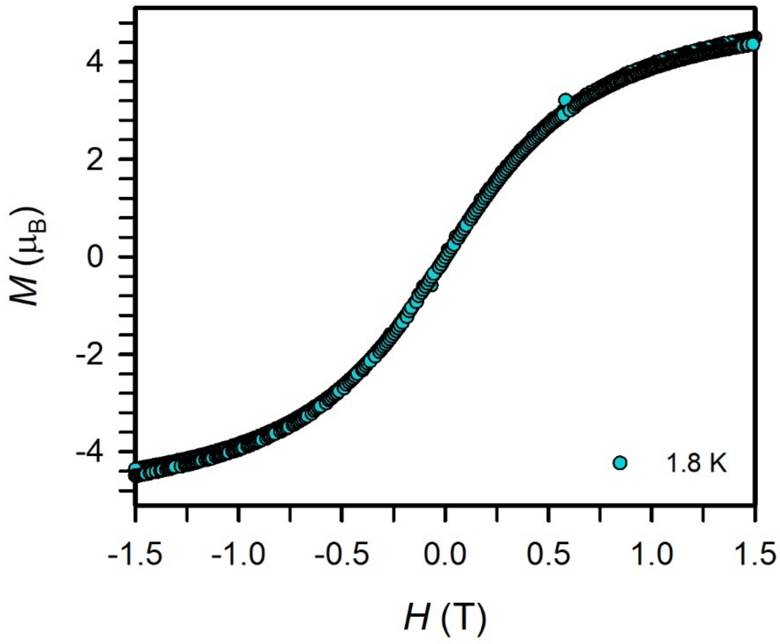

Figure S20. Plot of magnetization (*M*) vs dc magnetic field (*H*) at an average sweep rate of 100 Oe/s for **2** at 1.8 K.

***Ab initio* calculation**

The spin ground state of [(NHAr*)$_2$Dy] (**2**) was calculated via a series of Complete Active Space Self-Consistent Field (CASSCF) with the RIJCOSX approximation[7] as implemented in ORCA 5.0.4.[8,9] Scalar relativistic effects were accounted for with the second order Douglas-Kroll approach,[10,11] where the DKH-def2-SVP basis set was used for all atoms and C atoms of the peripheral Tripp groups,[12] DKH-def2-TZVP for Cl, N, and C atoms,[13] of the coordinating Tripp groups and SARC2-DKH-QZVP basis sets for the Dy atom.[14] Auxiliary basis sets for C, N and H were generated via the autoaux feature.[15] Finer integration grids (defgrid 3) with tight convergence criteria (energy convergence tolerance: 1e−07) were used throughout with disabled frozen core approximation. The calculations were carried out on the coordinates obtained from single-crystal XRD for [(NHAr*)$_2$Dy] (**2**), where all hydrogen positions were initially optimized via the TPSSh functional.[16,17] The most suitable active space was determined via a series of CASSCF calculations probing the 4*f* orbitals only (10,7), with additional 5*d* orbitals (10,12) and with additional 5*d* and 6*s* orbitals (10,13). We then probed the exclusion of the empty 5d orbitals from the (10,12) calculation in a (10,9) calculation. For these calculations 35 S = 3 states were considered for the state-averaged (SA) CASSCF calculation. The obtained hybrid orbitals are of primary ligand character and therefore the incorporation of ligand orbitals alongside 5*d* orbitals was probed in a (10,15) calculation. A final calculation was carried out on the (10,15) orbitals by considering 35 S = 3 and 35 S = 2 states.

Table S1. State-averaged occupation numbers of the optimized active orbitals obtained from different CASSCF calculations over 35 roots. The 4*f* orbital occupation numbers are in accordance with 9 electrons throughout. Since CASSCF natural orbitals are employed, the 4*f* and 5*d* orbitals are mixtures of different *f*/*d* orbitals, respectively, and thus, numbers were assigned rather than using a description of their atomic orbitals. Hybrid orbitals are indicated with {}.

Active space	State-averaged occupation numbers
(10,7)	7 4*f* (1.429)
(10,12)	7 4*f* (1.285), $\{5d,L\}_1$ (0.514), $\{5d,L\}_2$ (0.486),<br>$5d_1$ (0.001), $5d_2$ (0.001), $5d_3$ (0.001)
(10,9)	7 4*f* (1.285), $\{5d,L\}_1$ (0.514), $\{5d,L\}_2$ (0.486)
(10,13)[a]	7 4*f* (1.285), $\{5d,L\}_1$ (0.514), $\{5d,L\}_2$ (0.486)<br>$5f_1$ (0.001), $5f_2$ (0.001), $5f_3$ (0.001), $5f_4$ (0.001)
(10,15)	7 4*f* (1.284), $\{5d,L\}_1$ (0.514), $\{5d,L\}_2$ (0.314), $\{5d,L\}_3$ (0.172)<br>$5d_1$ (0.002), $5d_2$ (0.002), $5d_3$ (0.002), $5d_4$ (0.002), $5d_5$ (0.002)

[a]Active space composition changed during orbital optimization.

Table S2. Composition of active space orbitals after orbital optimization for Dy 5*d*, 6*s*, and 6*p* contributions.

Active Space	Hybrid orbitals	5*d* (%)	6*s* (%)	6*p* (%)
(10,7)	-	-	-	-
(10,12)	$\{5d,L\}_1$	17.5	0.7	0.3
	$\{5d,L\}_2$	6.2	0.0	0.4
	$5d_1$	91.6	0.0	0.0
	$5d_2$	88.	0.0	0.1
	$5d_3$	90.1	0.0	0.3
(10,9)	$\{5d,L\}_1$	17.5	0.7	0.3
	$\{5d,L\}_2$	6.2	0.0	0.4
(10,13)[a]	$\{5d,L\}_1$	14.5	0.5	0.2
	$\{5d,L\}_2$	10.9	0.1	1.3
(10,15)	$\{5d,L\}_1$	18.2	0.7	0.3
	$\{5d,L\}_2$	15.7	0.0	2.2
	$\{5d,L\}_3$	5.9	0.0	0.5
	$5d_1$	96.7	0.0	0.0
	$5d_2$	95.9	0.0	0.0
	$5d_3$	95.1	0.0	0.0
	$5d_4$	92.0	0.0	0.0
	$5d_5$	91.8	0.0	0.2

[a]Active space composition changed during orbital optimization.

Table S3. Relative spin-free energies for different CASSCF calculations for 35 roots of the high spin state of $[(NHAr^*)_2Dy]$ (**2**) $S = 3$ in cm^{-1}.

State	CAS (10,7)	CAS (10,12)	CAS (10,9)	CAS (10,13)[a]	CAS (10,15)
**1**	0.0	0.0	0.0	0.0	0.0
**2**	72.4	48.6	28.9	11.0	32.0
**3**	169.0	216.9	206.3	2394.5	250.2
**4**	215.1	367.2	358.9	2866.3	441.2
**5**	261.2	385.5	405.4	3832.0	490.5
**6**	340.2	629.5	639.2	4558.5	797.9
**7**	526.7	683.1	730.1	4681.4	917.9
**8**	542.1	1000.4	913.9	5164.2	1229.0
**9**	588.2	1000.9	934.5	5210.3	1250.6
**10**	627.7	1428.4	1229.5	5697.6	1652.7
**11**	649.6	1429.8	1237.9	5706.3	1659.3
**12**	697.9	7830.8	7836.4	5833.6	6833.0
**13**	783.5	7844.3	7876.0	5888.5	6910.2
**14**	16223.6	8068.0	8102.2	8144.7	6920.1
**15**	16311.4	8120.5	8106.9	8618.8	7104.7
**16**	16502.3	8213.6	8148.3	9586.7	7291.4
**17**	16511.1	8262.4	8199.1	10321.9	7513.4
**18**	16530.8	8300.5	8231.7	10411.9	7719.3
**19**	16618.6	9220.2	9345.2	10811.3	7825.1
**20**	16708.6	9254.4	9412.0	10943.0	7885.8
**21**	16803.0	9472.0	9440.8	10967.2	7964.8
**22**	25384.5	9542.8	9564.3	11044.0	8036.2
**23**	25555.6	9599.3	9592.4	11601.4	8221.2
**24**	25685.1	9754.1	9685.3	11625.6	8225.1
**25**	25687.3	9786.2	9766.6	11695.8	8284.7
**26**	25810.2	9904.2	9792.2	12097.4	8360.7
**27**	25836.6	9964.3	9836.3	12281.8	8363.3
**28**	25893.6	10209.5	10037.4	12496.9	8402.0
**29**	25935.3	10216.7	10062.9	13025.8	8429.3
**30**	26016.5	16983.0	16999.3	16623.0	9637.2
**31**	41469.8	17008.5	17015.2	16820.5	9703.1
**32**	41614.6	17055.7	17122.4	17391.2	9733.9
**33**	41669.5	17116.5	17141.1	17865.2	9882.5
**34**	42257.7	17178.9	17171.5	18045.2	9922.1
**35**	42345.5	17297.2	17211.7	18264.7	10079.1

[a]Active space composition changed during orbital optimization.

Table S4. Summary of single point energies obtained from different CASSCF calculations.

Active Space	Single Point Energy (H)
(10,7)	-15038.60802
(10,12)	-15038.78302
(10,9)	-15038.77702
(10,13)[a]	-15038.83289
(10,15)	-15038.80125

[a]Active space composition changed during orbital optimization.

Table S5. Relative energies of the high spin $S = 3$ and low spin $S = 2$ states obtained from the final CASSCF (10,15) calculation in cm^{-1}. The energy of the first state $S = 3$ state is 1036.6 cm^{-1} lower than the first $S = 2$ state.

State	$S = 3$	$S = 2$
**1**	0.0	0.0
**2**	32.0	59.9
**3**	250.2	62.1
**4**	441.2	169.8
**5**	490.5	315.6
**6**	797.9	501.3
**7**	917.9	743.7
**8**	1229.0	1037.5
**9**	1250.6	1046.6
**10**	1652.7	1316.2
**11**	1659.3	1340.6
**12**	6833.0	6095.5
**13**	6910.2	6259.5
**14**	6920.1	6377.4
**15**	7104.7	6563.7
**16**	7291.4	6576.0
**17**	7513.4	6944.1
**18**	7719.3	6973.6
**19**	7825.1	7324.7
**20**	7885.8	7386.3
**21**	7964.8	7553.1
**22**	8036.2	7590.1
**23**	8221.2	7764.8
**24**	8225.1	7831.8
**25**	8284.7	8035.1
**26**	8360.7	8043.5

**27**	8363.3	8091.9
**28**	8402.0	8137.1
**29**	8429.3	8169.1
**30**	9637.2	8749.4
**31**	9703.1	8787.6
**32**	9733.9	8886.3
**33**	9882.5	8970.7
**34**	9922.1	9045.6
**35**	10079.1	9243.2

Single Crystal X-ray Diffraction

Single crystal data was collected on XtaLAB Synergy, Dualflex, Hypix diffractometer using CuK_{α} or MoK_{α} radiation. Data collection was done at 100 K under a continuous flow of liquid nitrogen. In Olex2 program, crystal structures were solved with ShelXT solution using intrinsic phasing and refined with the SheXL refinement package using least squares minimization.[18,19] All hydrogens are refined anisotropically. All crystals were stable at room temperature for mounting.

An Alert B is present in the $Dy(NHAr^{*})_2NC$ (**3**) checkcif file due to a terminal carbon in isocyanide. Thermal ellipsoids are drawn with a 50% probability level. Orange, blue, gray, and white spheres represent dysprosium, nitrogen, carbon, and hydrogen atoms, respectively.

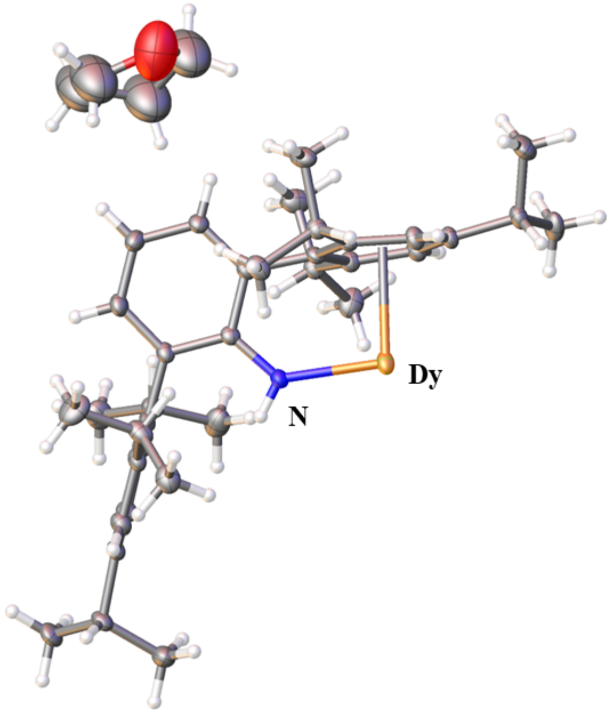

Figure S21. Asymmetric unit of Dy(NHAr*)$_2$ (**2**) recrystallized from THF. Complex **2** crystallizes with one molecule of THF in the lattice. Ellipsoids at the 30% probability level.

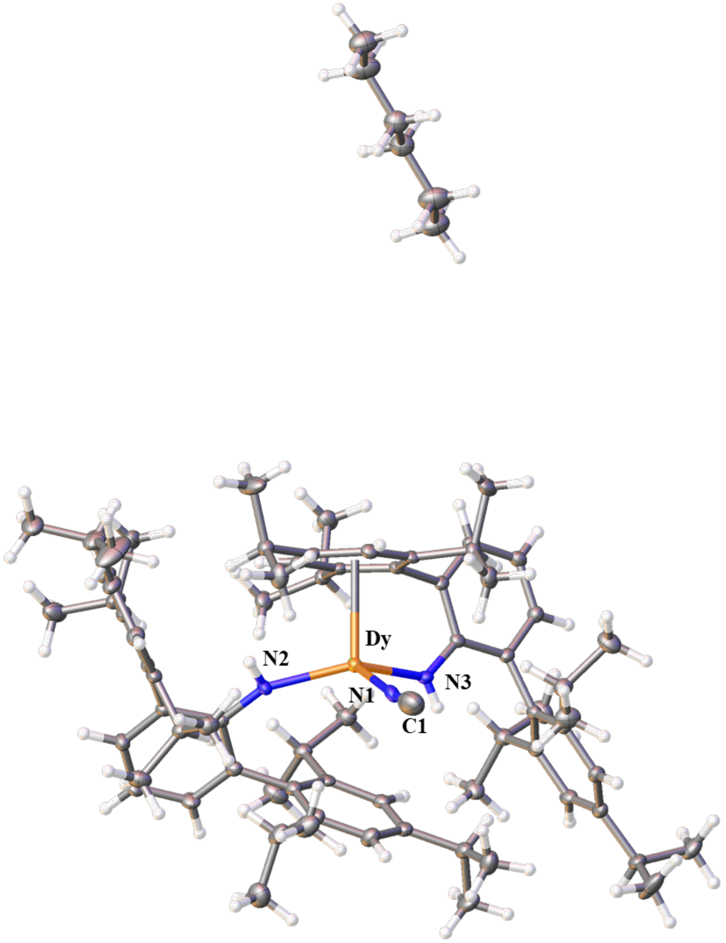

Figure S22. Structure of Dy(NHAr*)$_2$NC (**3**) recrystallized from *n*-hexane. Half a molecule of *n*-hexane is in the lattice per molecule of **3**. Ellipsoids at the 30% probability level.

We tried to solve the structure by exchanging the positions of C1 to N1 and N1 to C1. Analysis of theoretical cyanide structure gave us poor refinement values R_1 = 3.78% and wR_2 = 9.14%. Additionally, we tried assigning variable percentages of atom occupancy to N and C; however, all attempts increased refinement parameters.

Table S6. Thermal parameters for carbon and nitrogen atoms in $Dy(NHAr^*)_2NC$ (**3**) vs $Dy(NHAr^*)_2CN$ (**3’**) crystal structure.

		C	N
$Dy(NHAr^*)_2NC$	U11	0.0342(17)	0.0155(9)
	U22	0.0393(17)	0.0169(9)
	U33	0.065(3)	0.0284(12)
$Dy(NHAr^*)_2CN$	U11	0.0180(12)	0.0277(18)
	U22	0.0193(12)	0.0328(17)
	U33	0.0111(10)	0.207(6)

Table S7. Metric data from the crystal structures of $Dy(NHAr^*)_2$ (**2**) $Dy(NHAr^*)_2NC$ (**3**),

Complex	$Dy(NHAr^*)_2$ (**2**)	$Dy(NHAr^*)_2NC$ (**3**)
Dy–N<br><br>Dy–N1	2.275(2)	2.2182(19)<br>2.2366(19)<br>2.372(2)
N–C_{ipso}	1.378(3)	1.392(3)<br>1.392(3)
N1–C1		1.025(4)
Dy-Ar_{Cent}	2.46007(9)	2.5090(2)
Dy–N–C_{ipso}	130.38(16)	147.73(16)<br>133.09(15)
N–Dy–N	102.07(11)	136.80(7)<br>104.26(7)<br>104.59(7)
N–Dy–Cl		
A_{rcent}–Dy–N	95.48(5)<br>113.07(5)	104.24(5)<br>93.83(5)<br>112.02(5)
Dy–N–$C_{terminal(NC)}$		174.6(2)
Cent–Dy–Cent	134.399(7)	
η^6-C_{Ar}–C_{Ar}	1.433(4), 1.402(3),<br>1.411(4), 1.389(4),<br>1.419(4), 1.415(4)	1.414(3), 1.421(3),<br>1.406(3), 1.406(3),<br>1.402(3), 1.393(3)
Average C_{Ar}–C_{Ar} bond	1.411(4)	1.407(3)

Structure Analysis of [Dy(NHAr)$_2$][Dy(NHAr*)$_2$P$_4$] (4)*

A metallic intense orange cube-shaped crystal with dimensions 0.26×0.14×0.11 mm^3 was mounted on a nylon loop with paratone oil. Data were collected using a XtaLAB Synergy, Dualflex, HyPix diffractometer equipped with an Oxford Cryosystems 800 low-temperature device, operating at T = 100.00(10) K.

Data were measured using w scans using Cu K$_a$ radiation (micro-focus sealed X-ray tube, 50 kV, 1 mA). The total number of runs and images was based on the strategy calculation from the program CrysAlisPro system (CCD 43.119a 64-bit (release 08-04-2024)). The achieved resolution was Q = 80.287.

Cell parameters were retrieved using the CrysAlisPro 1.171.43.119a (Rigaku OD, 2024) software and refined using CrysAlisPro 1.171.43.119a (Rigaku OD, 2024) on 23351 reflections, 22% of the observed reflections. Data reduction was performed using the CrysAlisPro 1.171.43.119a (Rigaku OD, 2024) software, which corrects for Lorentz polarization. The final completeness is 100.00 out to 80.287 in Q CrysAlisPro 1.171.43.119a (Rigaku Oxford Diffraction, 2024). Numerical absorption correction is based on Gaussian integration over a multifaceted crystal model. Empirical absorption correction using spherical harmonics is implemented in the SCALE3 ABSPACK scaling algorithm.

The structure was solved in the space group $Pca2_1$ (# 29) by using dual methods using the ShelXT 2018/2 structure solution program.[18] The structure was refined by Least Squares ShelXL incorporated in the Olex2 software program.[19] All non-hydrogen atoms were refined anisotropically. Hydrogen atom positions were calculated geometrically and refined using the riding model. The ether molecules were not clearly identified by the residual electron density. Program BYPASS in Olex2 suggested there are three ether molecules per asymmetric unit cell.

Therefore, we did our best to model and refine these molecules isotropically. Once in location, hydrogens were added, and the molecules were restrained with the AFIX 1 command, as these molecules would not converge. Using the BYPASS method in Olex2, we calculated the solvent as three ether molecules per asymmetric unit cell.

```
_smtbx_masks_void_probe_radius    1.2
_smtbx_masks_void_truncation_radius 1.2
loop_
 _smtbx_masks_void_nr
 _smtbx_masks_void_average_x
 _smtbx_masks_void_average_y
 _smtbx_masks_void_average_z
 _smtbx_masks_void_volume
 _smtbx_masks_void_count_electrons
 _smtbx_masks_void_content
 1  -0.192  -0.417  0.769  869.9  150.8  '3 C5H10O'
 2   0.192  -0.416  0.269  869.9  143.7  '3 C5H10O'
 3   0.308  -0.597  0.769  869.9  150.8  '3 C5H10O'
 4   0.692  -0.289  0.269  869.9  143.7  '3 C5H10O'
```

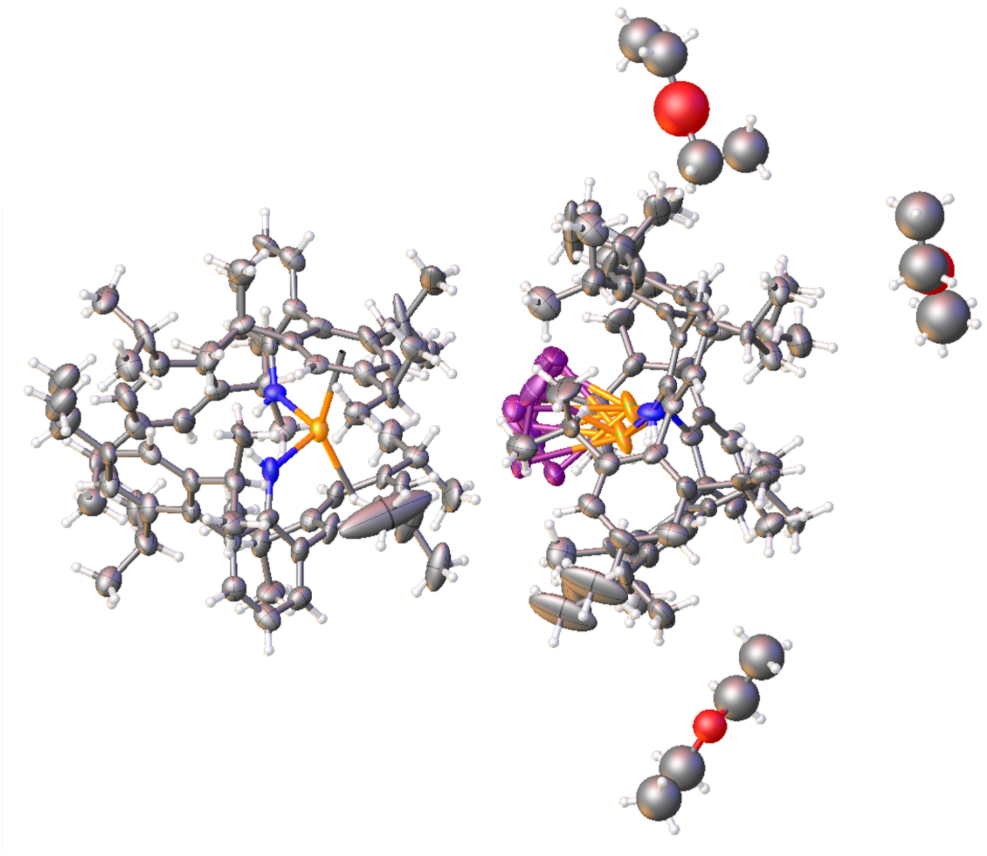

Figure S23. Structure of [Dy(NHAr*)$_2$][Dy(NHAr*)$_2$P$_4$] **4** recrystallized from diethyl ether. Three molecules of diethyl ether are in the lattice per molecule of **4**. Ellipsoids at the 30% probability level.

This is the best of crystals grown from various solvents and various data collections. All crystals had poor diffraction, and they must be used immediately once removed from the glove box in oil. This crystal was a racemic twin and showed significant disorder. Initial trials attempted to put the two P_4 units in a butterfly orientation, but this proved to be very unsuccessful. The Dy atom was modeled disordered, and the occupancy was refined to that of the P_4 unit best, where the largest occupancy went with the P_4 in the butterfly orientation. These were then locked as one free variable (70:30). It was anticipated that the rest of the ligand would have some portion of disorder, but so

far attempts to model this molecule closer to a full molecule disorder have failed, leaving what may be some distorted Dy–N bond lengths.

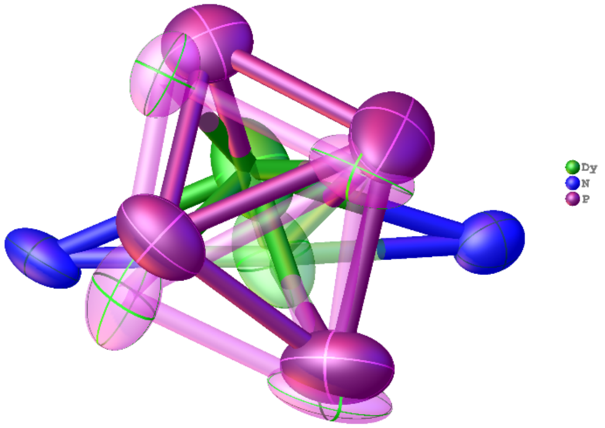

Figure showing the two orientation, minor one is 50% translucent to better show the orientation.

There is a single formula unit in the asymmetric unit, which is represented by the reported sum formula. In other words, Z is 4, and Z' is 1. The moiety formula is $C_{72}H_{100}DyN_2P_4$,$C_{72}H_{100}DyN_2$, 3($C_4H_{10}O$).

The Flack parameter was refined to 0.513(12). Determination of absolute structure using Bayesian statistics on Bijvoet differences using the Olex2 results in 0.043(4). The chiral atoms in this structure are: C7A(R), C8A(R), C9A(R), C11A(S), C12A(S), C43A(S), C44A(R), C45A(R), C47A(S), C48A(S). Note: The Flack parameter is used to determine the chirality of the crystal studied, and the value should be near 0. A value of 1 means that the stereochemistry is wrong, and the model should be inverted. A value of 0.5 means that the crystal consists of a racemic mixture of the two enantiomers. (Ellipsoids at the 30% probability level.)

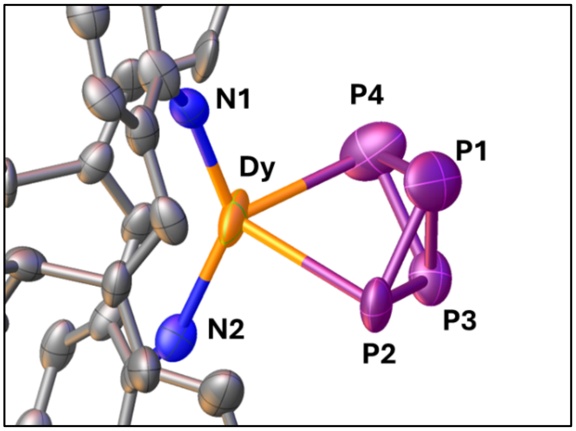

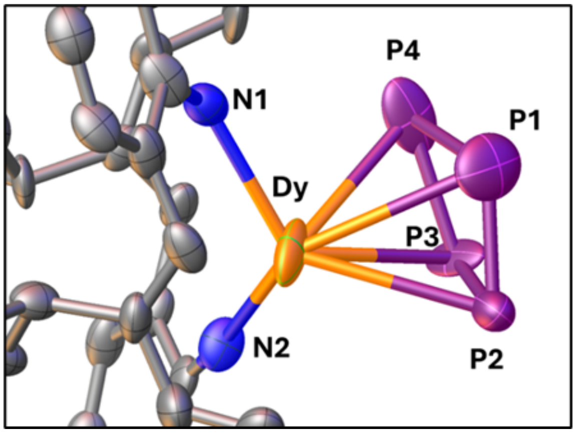

Bond Lengths (Å)/ Angles (°)	Butterfly-P_4^{2-}	Cyclo-P_4^{2-}
Dy–N1<br>Dy–N2	2.190 (13)<br>2.213(14)	2.537(16)<br>2.254(12)
Dy–P2<br>Dy–P4<br>Dy–P1<br>Dy–P3	2.811(11)<br>2.659(18)<br>3.279(15)<br>3.320(7)	2.965(12)<br>3.01(3)<br>3.01(4)<br>3.006(16)
P2–P3<br>P3–P4<br>P4–P1<br>P1–P2<br>P1–P3<br>P2–P4	2.182(13)<br>2.17(2)<br>2.182(19)<br>2.112(16)<br>2.296(9)<br>3.21(2)	2.13(2)<br>2.12(3)<br>2.14(4)<br>2.01 (3)<br>2.45(3)<br>3.37(13)
Dy–Cent	3.638(12), 3.304(12)	4.109(12), 2.984(11)
Dy–P2–P1	82.2(5)	71.4(7)
Dy–P4–P1	84.6(7)	69.0(12)
P3–P2–P1	64.6(5)	72.4(10)
P3–P4–P1	63.7(6)	70.1(11)
P4–P3–P2	95.2(5)	104.9(10)
P2–P1–P4	96.9(6)	108.3(14)
N1–Dy–N2	136.5(5)	117.1(5)

Figure S24. (top) Zoom-in for P_4^{2-} butterfly (left) and planar (right) form. (bottom) Structural metrics for **4** from single-crystal x-ray diffraction.

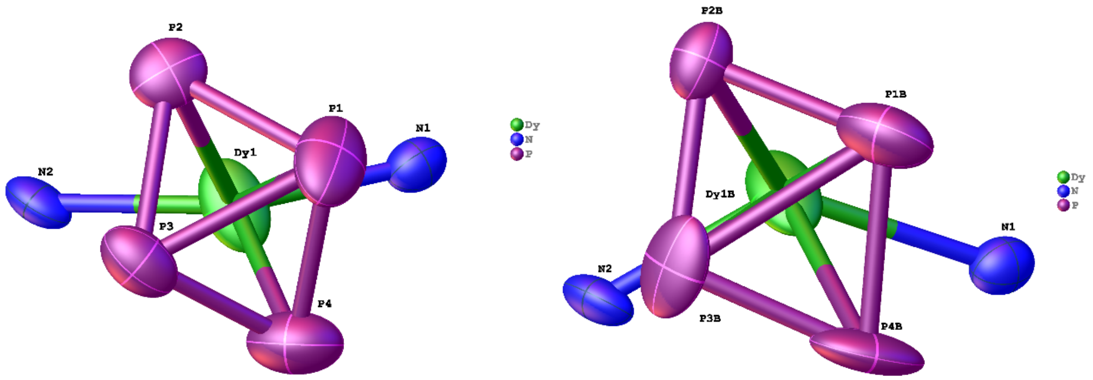

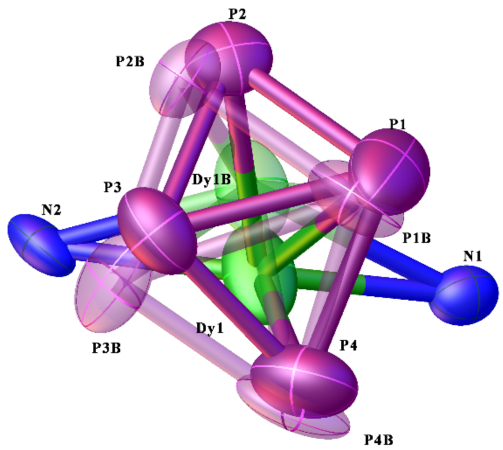

Various Drawings showing relative orientation of the two components. The P4 units are essentially centered over the Dy atom as expected.

Table S8. Comparison of counter cation $[Dy(NHAr^*)_2]^+$ from structure **4** to $[Dy(NHAr^*)_2][BArF_{24}]$ structure.

Complex	**$[Dy(NHAr^*)_2]$**$[Dy(NHAr^*)_2P_4]$ (**4**)	**$[(NHAr^*)_2Dy]$**$[BArF_{24}]$
Dy–N	2.235(15), 2.217(16)	2.222(2)
N–C_{ipso}	1.40(2)	1.394(4)
Dy-Ar_{Cent}	2.534(9), 2.512(9)	2.49669(3)
Dy–N–C_{ipso}	132.0(9), 131.9(10)	131.50(19)
N–Dy–N	108.8(5)	131.72(13)
Ar_{cent}–Dy–N	109.3(3), 94.4(5)	94.29(6)
Cnt–Dy–Cnt	140.15	152.5427(5)

Table S9. Crystallographic data and structural refinement of $Dy(NHAr^*)_2Cl$ (**1**), $Dy(NHAr^*)_2$ (**2**), and $Dy(NHAr^*)_2NC$ (**4**).

**Compound**	$[Dy(NHAr^*)_2][Dy(NHAr^*)_2P_4]$ (**4**)
Formula	$C_{156}H_{230}Dy_2N_4O_3P_4$
$D_{calc.}$/ g cm^{-3}	1.169
m/mm^{-1}	6.006
Formula Weight	2658.31
Color	metallic intense orange
Shape	cube-shaped
Size/mm^3	0.26×0.14×0.11
T/K	100.00(10)
Crystal System	orthorhombic

Flack Parameter	0.513(12)
Hooft Parameter	0.043(4)
Space Group	$Pca2_1$
a/Å	25.2032(3)
b/Å	21.9756(3)
c/Å	27.2612(4)
a/°	90
b/°	90
g/°	90
V/Å^3	15098.8(4)
Z	4
Z'	1
Wavelength/Å	1.54184
Radiation type	Cu K_a
Q_{min}/°	2.668
Q_{max}/°	80.287
Measured Refl's.	108558
Indep't Refl's	28739
Refl's I≥2 *s*(I)	19142
R_{int}	0.0938
Parameters	1419
Restraints	604
Largest Peak	1.844
Deepest Hole	-2.125
GooF	1.080
wR_2 (all data)	0.2785
wR_2	0.2427
R_1 (all data)	0.1207
R_1	0.0857

DFT Calculations

DFT calculations were carried out using Gaussian 16 (B01 and C01).[20,21] The starting coordinates for the geometry optimizations were taken from the structure found from X-ray diffraction. Geometry optimization and frequency calculations were performed using the TPSSH hybrid functional[16,22] and def2-SVP basis set[12] on C, N, H, and ECP55MWB pseudopotential[23,24] on the dysprosium atom with Grimme's dispersion correction GD3.[25,26] The colors turquoise, blue, grey, and white spheres represent dysprosium, nitrogen, carbon, and hydrogen atoms, respectively.

First, the DFT calculations were performed Dy(NHAr*)$_2$ (**2**) to identify the distribution of the unpaired electron density. The comparison of optimized bond lengths and bond angles is shown below. The theoretically obtained structure is consistent with the crystal structure. Further, the HOMO, LUMO, and electron charge density map looks similar to our previously reported Y(II) complex.[5] This suggests that the ground state electronic configuration for **2** is $4f^9 5d^1$, where the unpaired electron is delocalized majorly over the arene system.

Table S10. Structural comparisons between crystallographically obtained geometry of **2** and optimized geometry using DFT calculations. Shown here are some characteristic lengths (Å) and angles (˚).

Atoms	Experimental Distance (Å)/Angle (˚)	Theoretical Distance (Å)/Angle (˚)
Dy–N	2.275(2)	2.316
Dy-Ar$_{Cent}$	2.460(1)	2.476
Cent–Dy–Cent	134.399(7	135.17
Dy–N–C$_{ipso}$	130.38(16)	130.01
A$_{rcent}$–Dy–N	95.48(5)<br>113.07(5)	95.40<br>112.31
Average C$_{Ar}$–C$_{Ar}$ bond	1.411(4)	1.423

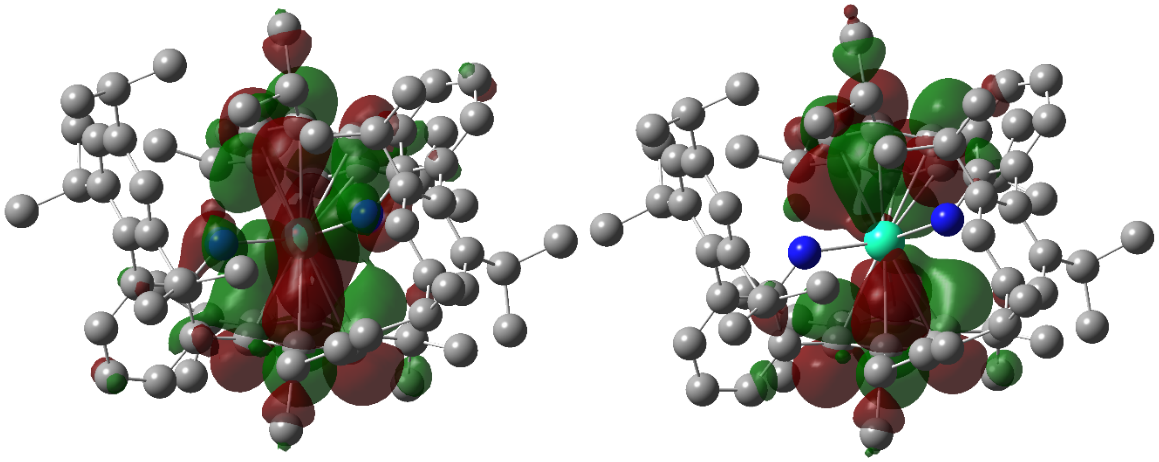

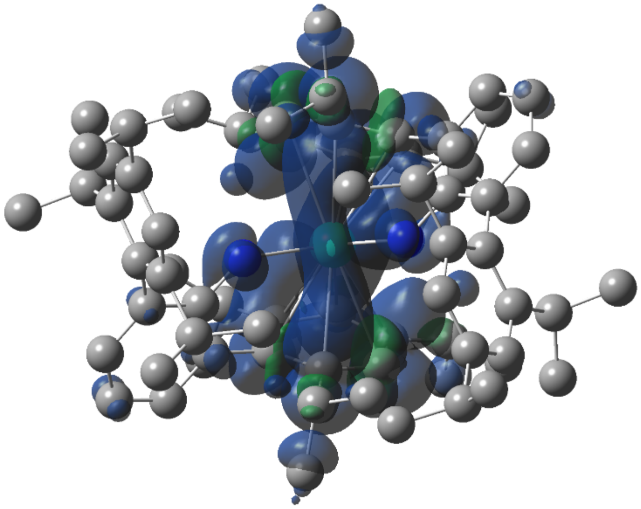

Figure S25. HOMO (top, left), LUMO (top, right), and spin density map (bottom) of **2**.

The preference for the formation of dysprosium isocyanide over hypothetical cyanide is discussed here. The structure obtained from DFT calculations is consistent with the single-crystal structure **3** (Table S6). The optimized geometries of **3** and **3’** are shown below.

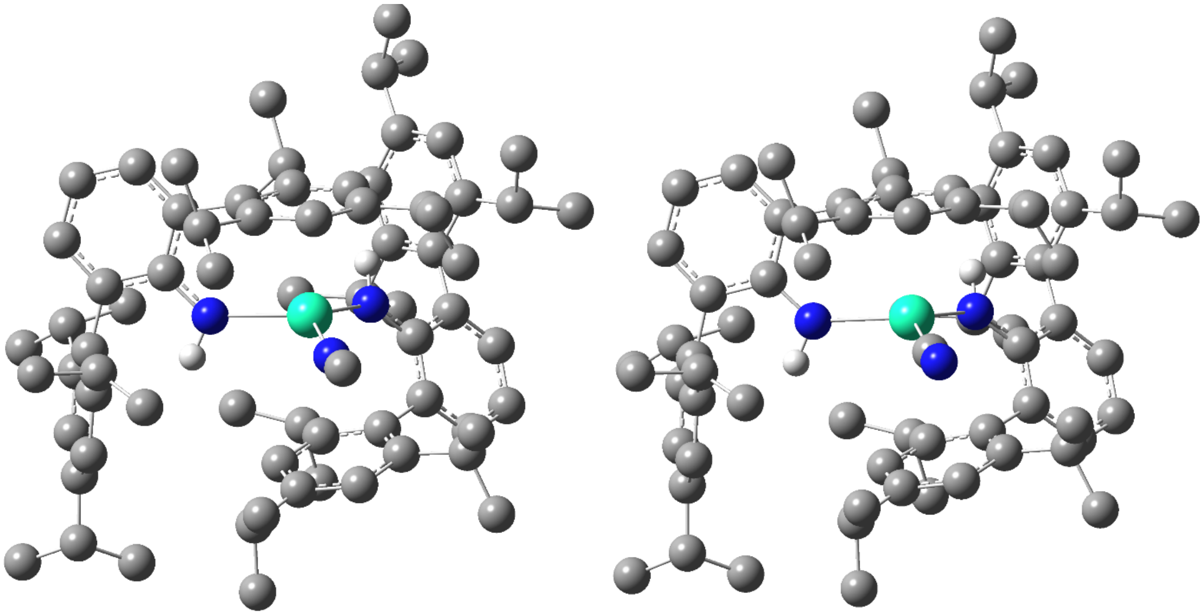

Figure S26. Optimized structures of Dy(NHAr*)$_2$NC (**3**) (left) and Dy(NHAr*)$_2$CN (**3'**) (right). All hydrogens except N–H hydrogens are removed for clarity.

Table S11. Structural comparisons between crystallographically obtained geometry of Dy(NHAr*)$_2$NC (**3**) and optimized geometry using DFT calculations. Shown here are some characteristic bond lengths (Å) and angles (˚).

Atoms	Experimental Distance (Å)/Angle (˚)	Theoretical Distance (Å)/Angle (˚)
Dy–N2, N3	2.2182(19)<br>2.2366(19)	2.27562<br>2.28537
Dy-N1	2.372(2)	2.332
Dy-Ar$_{Cent}$	2.5178(2)	2.570
N1–C1	1.025(5)	1.184
Dy–N1–C1	174.6	169.86
Dy–N–C$_{ipso}$	147.73(16)<br>133.10(15)	136.97<br>134.68

The ground energy difference between Dy(NHAr*)$_2$NC (**3**) and hypothetical Dy(NHAr*)$_2$CN (**3'**) is ~2.5 kcal/mol. Further frequency calculation gave us ν_{NC} stretch for **3** at 2131.5 cm$^{-1,}$ which is relatively good agreement with the experimental value (2052 cm^{-1}). The calculated ν_{NC} for the hypothetical Dy(NHAr*)$_2$CN (**3'**) is 2217.6 cm^{-1} higher than isocyanide due to the lone pair bond weakening effect discussed in our previous study.[5]

Additionally, ground state energy calculations were performed on [(NHAr*)$_2$Dy–P$_4$]$^-$ to confirm the charge distribution. The [(NHAr*)$_2$Dy]$^+$ was excluded from the calculations to improve computational efficiency and convergence time. The optimized geometry for butterfly and pseudo planar dysprosium P_4^{2-} structures are shown in Figure S30. The optimized geometry is in close approximation with the crystal structure.

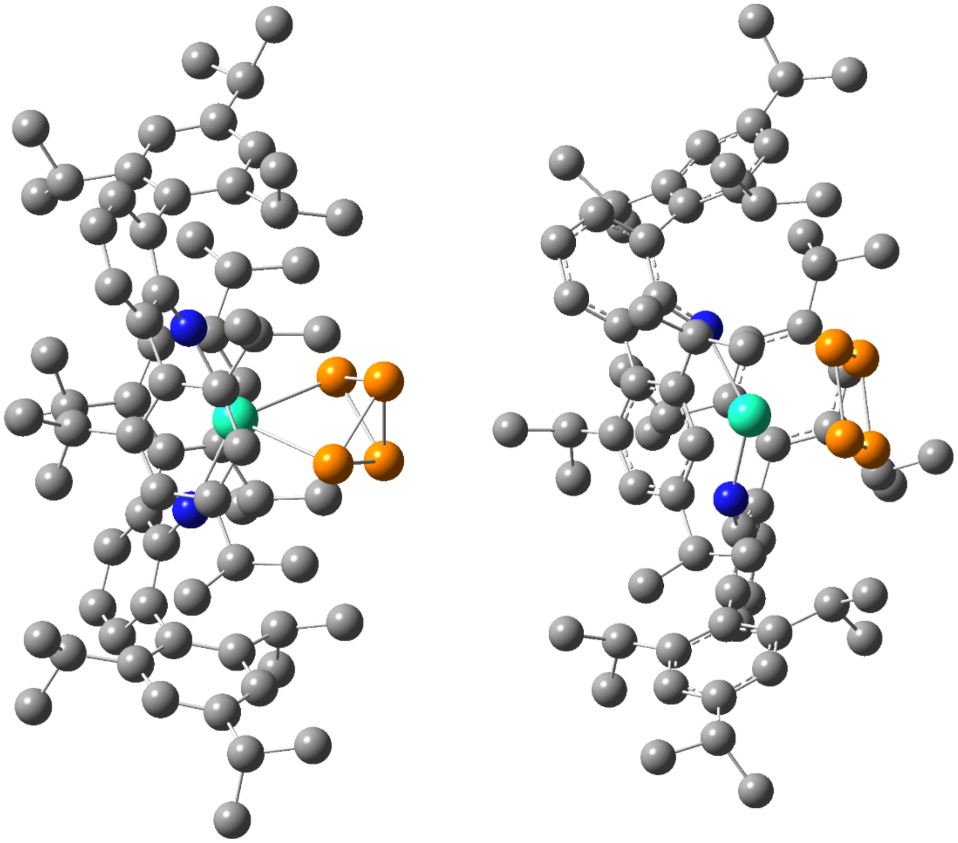

Figure S27. Optimized structures of butterfly-$[(NHAr^*)_2Dy{-}P_4]^-$ (left) and pseudo-planar $[(NHAr^*)_2Dy{-}P_4]^-$ (right). All hydrogens except N–H hydrogens are removed for clarity.

Table S12. Structural comparisons between crystallographically obtained geometry of butterfly- and pseudo planar-$[(NHAr^*)_2Dy{-}P_4]^-$, and optimized geometry using DFT calculations. Shown here are some characteristic bond lengths (Å) and angles (˚).

Bond Lengths (Å)/ Angles (°)	Experimental Butterfly P_4^{2-}	Theoretical Butterfly P_4^{2-}	Experimental Pseudo planar P_4^{2-}	Theoretical Pseudo planar P_4^{2-}
Dy–N1	2.190 (13)	2.319	2.537(16)	2.331
Dy–N2	2.213(14)	2.315	2.254(12)	2.2331
Dy–P2	2.811(11)	2.779	2.965(12)	2.989
Dy–P4	2.659(18)	2.775	3.01(3)	2.994
Dy–P1	3.279(15)	3.467	3.01(4)	2.966
Dy–P3	3.320(7)	3.466	3.006(16)	2.95

P2–P3<br>P3–P4<br>P4–P1<br>P1–P2<br>P1–P3<br>P2–P4	2.182(13)<br>2.17(2)<br>2.182(19)<br>2.112(16)<br>2.296(9)<br>3.21(2)	2.27<br>2.27<br>2.27<br>2.27<br>2.16<br>3.36	2.13(2)<br>2.12(3)<br>2.14(4)<br>2.01 (3)<br>2.45(3)<br>3.37(13)	2.17<br>2.18<br>2.17<br>2.18<br>3.07<br>3.06
Dy–Cent	3.638(12),<br>3.304(12)	3.513<br>3.485	4.109(12),<br>2.984(11)	3.96, 2.88
P3–P2–P1	64.6(5)	56.67	72.4(10)	89.9
P3–P4–P1	63.7(6)	56.69	70.1(11)	90.1
P4–P3–P2	95.2(5)	95.38	104.9(10)	89.9
P2–P1–P4	96.9(6)	95.43	108.3(14)	90.1
N1–Dy–N2	136.5(5)	128.20	117.1(5)	123.3

The frequency calculations were done to obtain thermochemical data on both isomers of the $[(NHAr^*)_2Dy(P_4)]^-$ structure. The sum of electronic and thermal free energies for butterfly and pseudo planar $[(NHAr^*)_2Dy(P_4)]^-$ are –4312.944645 and –4312.946343 Hartree/particle, respectively. This suggests that the planar binding mode of the P_4 unit is thermodynamically more stable (~1 kcal/mol) than the butterfly binding mode. This small energy difference indicates that both forms will coexist as obtained from the crystal structure. As expected, HOMO and LUMO are heavily located on anionic phosphorus for both forms of $[(NHAr^*)_2Dy(P_4)]^-$ structure.

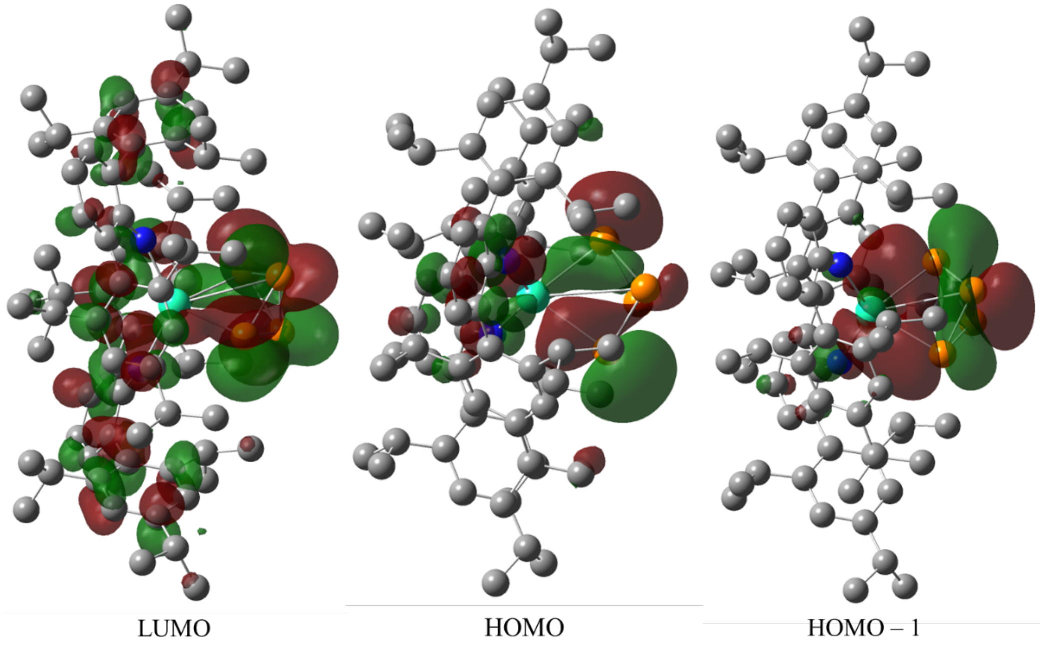

Figure S28. Frontier molecular orbitals for butterfly-[(NHAr*)$_2$Dy–P$_4$]$^-$ structure.

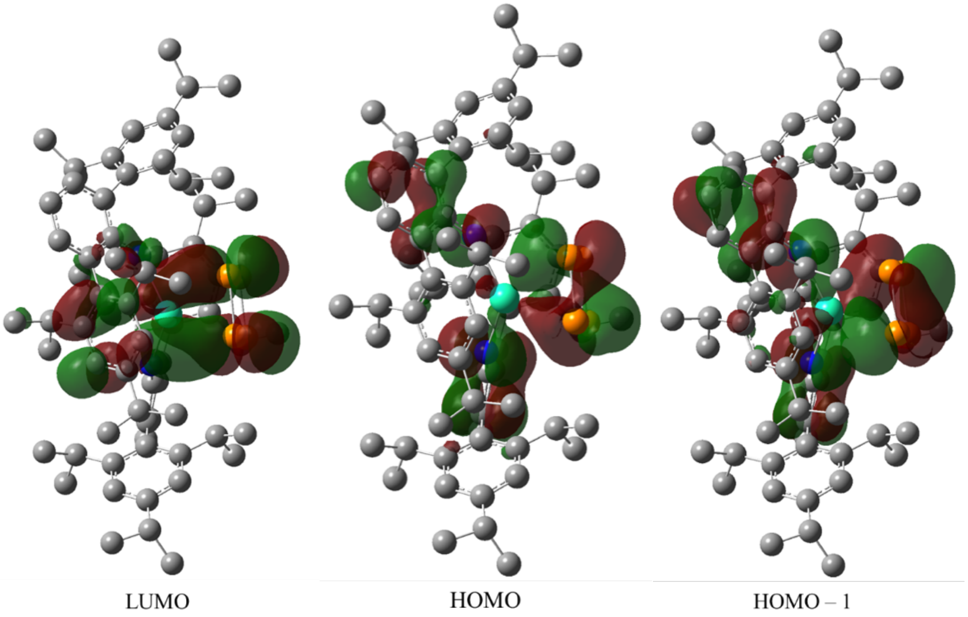

Figure S29. Frontier molecular orbitals for cyclo-[(NHAr*)$_2$Dy–P$_4$]$^-$ structure.

We tried to locate the transition state for interconversion of butterfly-P_4^{2-} complex and cyclo-P_4^{2-}. For this, we used ground state structures of H_2Dy-(b/c-P_4^{2-}) and $(PhNH)_2$Dy-(b/c-P_4^{2-}). The transition state with one negative frequency was obtained and the results are summarized below. The energy of butterfly form was set to zero for comparison.

Table S13. Relative enthalpies (TPSSh/def2-SVP) of butterfly and cyclo-binding mode of P_4^{2-}unit with transition state.

Structure	Enthalpy	b-P_4^{2-}	c-P_4^{2-}	TS
H_2Dy-(b/c-P_4^{2-})	Hartrees	-1403.416714	-1403.418120	-1403.368495
	kcal/mol	0	-0.9	30.3
$(PhNH)_2$Dy-(b/c-P_4^{2-})	Hartrees	-1975.780903	-1975.78332	-1975.733008
	kcal/mol	0	-1.5	30.0

Optimized Geometry Coordinates

Dy(NHAr)₂ (**2**)*

N -1.7364894 -0.3321525 0.4846972

N 1.7745051 -0.0892693 -0.483933

C -0.8911348 -1.8529015 2.5660277

C 1.1796816 -1.7673284 -2.5313061

C -0.8669007 -3.1802928 2.0854625

C 1.3592052 -3.0700948 -2.021265

C 0.3894677 -3.7637695 1.741965

C 0.2091448 -3.8324569 -1.6542971

C 1.63017 -3.1348801 2.0799943

C -1.115034 -3.4121984 -2.0070515

C 1.5750336 -1.8187137 2.5443729

C -1.2637558 -2.1165641 -2.5092524

C 0.347941 -1.1330368 2.7441941

C -0.1611357 -1.2489118 -2.7217993

H	-2.0832554	0.3433121	-0.1993393
C	-2.5004425	-0.2631815	1.6223981
H	2.0174415	0.6380726	0.1905843
C	2.5264602	0.0678389	-1.6203631
C	-2.1545662	-1.086148	2.7373574
C	2.3126375	-0.820184	-2.7185161
C	-2.0939277	-4.0759039	1.9354002
C	2.7095842	-3.7617267	-1.8497682
H	0.399397	-4.794841	1.3820922
H	0.359719	-4.8432612	-1.2692398
C	2.9161261	-3.9394438	1.9858183
C	-2.2609239	-4.4034157	-1.8906209
H	2.5020084	-1.2914499	2.7720363
H	-2.261781	-1.7498933	-2.7478761
C	0.3508594	0.2610952	3.3603686
C	-0.3594059	0.1109653	-3.3818603
C	-3.6191272	0.6126867	1.7673815
C	3.5077436	1.0931871	-1.7753092
C	-2.9130688	-1.0767758	3.9083031
C	3.0839078	-0.7307862	-3.8780446
H	-1.7809448	-4.8644127	1.2271833
C	-2.3902068	-4.7717377	3.2773475
C	-3.3611681	-3.4299084	1.3536952
H	2.5179906	-4.5731838	-1.1244886
C	3.1156226	-4.4346227	-3.1744663
C	3.8601283	-2.9167803	-1.2800202
H	2.9637685	-4.3557631	0.9629047
C	2.8654377	-5.1329471	2.9622626
C	4.182881	-3.1156096	2.2247813
H	-2.2455574	-4.7978145	-0.8578903

C	-2.0256549	-5.5985949	-2.8374729
C	-3.6400483	-3.7918916	-2.1463303
H	-0.6786976	0.6410864	3.2971776
C	0.7094755	0.158623	4.855063
C	1.2501738	1.2674304	2.6346029
H	0.4955817	0.7393622	-3.0888526
C	-0.3094662	-0.0549216	-4.9140716
C	-1.6368724	0.8453571	-2.9651984
C	-4.3728384	0.5845897	2.9472192
C	-3.8741866	1.6142258	0.6853269
C	4.270025	1.1522392	-2.9481405
C	3.6002977	2.1472885	-0.7173365
H	-2.604697	-1.7059222	4.7477443
C	-4.0460905	-0.2606314	4.015668
H	2.8916102	-1.4241679	-4.7012669
C	4.0859715	0.2405329	-3.99598
H	-1.4986612	-5.287891	3.6665769
H	-3.1958921	-5.5138371	3.1549085
H	-2.7166026	-4.0340686	4.0262643
H	-3.9183357	-2.8581139	2.1068497
H	-4.0248668	-4.2171568	0.9622991
H	-3.114168	-2.742369	0.5315105
H	2.314844	-5.0887607	-3.5533649
H	4.0236652	-5.0430365	-3.032525
H	3.3310906	-3.6736601	-3.9398321
H	4.3304338	-2.2853059	-2.0446119
H	4.6325422	-3.5848753	-0.8668895
H	3.5060398	-2.2551704	-0.475989
H	2.8136499	-4.7744071	4.0031377
H	3.7695779	-5.7537898	2.8566964

H	1.9893736	-5.7741455	2.7823276
H	4.234879	-2.2448915	1.5581767
H	5.0765488	-3.7327294	2.04549
H	4.231595	-2.7534186	3.2646229
H	-2.0302349	-5.2622007	-3.8869784
H	-2.8219643	-6.3500418	-2.7132666
H	-1.0602542	-6.0906477	-2.6453302
H	-3.8271906	-2.9226316	-1.502306
H	-4.4275367	-4.53521	-1.948689
H	-3.7435105	-3.4684493	-3.1949175
H	0.0185868	-0.5204041	5.378583
H	0.6507731	1.1484183	5.3363677
H	1.7344635	-0.2257674	4.9916019
H	2.3022445	0.944831	2.5876741
H	1.2321819	2.2371986	3.1534298
H	0.8931794	1.4395456	1.6094019
H	0.6302049	-0.5307648	-5.2297745
H	-0.3816918	0.9253521	-5.4129558
H	-1.1472559	-0.6815686	-5.2632802
H	-2.5491233	0.2853783	-3.2228925
H	-1.6981746	1.8124614	-3.4851303
H	-1.6580761	1.0569691	-1.8878451
H	-5.2254878	1.263692	3.0354993
C	-3.2871066	2.9004814	0.7920719
C	-4.6263556	1.2745569	-0.4653576
H	5.0067031	1.9544197	-3.0492472
C	2.8085713	3.3149403	-0.8464403
C	4.4059349	1.9667356	0.433385
H	-4.6460941	-0.2585379	4.9277401
H	4.6908825	0.3076457	-4.9023369

C	-3.4805584	3.8253282	-0.2429495
C	-2.5029512	3.3021711	2.0380126
C	-4.7945468	2.2348313	-1.4739241
C	-5.276065	-0.1023354	-0.5709898
C	2.8521932	4.2857469	0.1639739
C	1.9061378	3.5315178	-2.0567074
C	4.4247064	2.969949	1.4133344
C	5.2625657	0.712167	0.5757225
H	-3.0320376	4.8186755	-0.1546101
C	-4.2318165	3.5140716	-1.3835314
H	-2.2513146	2.3749472	2.5754531
C	-3.3885488	4.147885	2.9706748
C	-1.1832678	4.0213494	1.7299994
H	-5.3779554	1.9862546	-2.3639187
H	-4.5924781	-0.8139484	-0.0766
C	-6.609559	-0.1326016	0.1990941
C	-5.489271	-0.5794287	-2.0129379
H	2.2367133	5.1842382	0.0583923
C	3.657621	4.1365753	1.2993882
H	2.0273607	2.658528	-2.7148504
C	2.3297435	4.7712422	-2.8609604
C	0.4252851	3.59771	-1.6554767
H	5.0500896	2.8455609	2.300938
H	4.7040532	-0.108422	0.0929007
C	6.5932045	0.8719516	-0.1826355
C	5.5276631	0.3092078	2.0321477
C	-4.4382459	4.5357079	-2.4930283
H	-2.8472962	4.4050569	3.8962636
H	-4.3038939	3.6034131	3.247555
H	-3.689535.0870302	2.4768162	

H	-1.3446955	4.9895845	1.2279792
H	-0.536804	3.4091746	1.0872549
H	-0.6377457	4.2254673	2.6645503
H	-6.4628704	0.1337553	1.2550893
H	-7.0587442	-1.1389138	0.1588504
H	-7.3243656	0.5820818	-0.2412128
H	-6.2393319	0.0332403	-2.5389582
H	-5.8577869	-1.616807	-2.0184081
H	-4.555282	-0.5435298	-2.5942468
C	3.7096129	5.2112283	2.3761983
H	1.6998377	4.8832462	-3.7588194
H	3.3794538	4.6911188	-3.1842184
H	2.2296097	5.692754	-2.2634782
H	0.2167219	4.4675726	-1.0125505
H	0.1278821	2.690851	-1.1082887
H	-0.2147129	3.6825795	-2.5471615
H	6.4184318	1.0859141	-1.246358
H	7.1940658	-0.0501101	-0.1128806
H	7.1821903	1.7007033	0.2438477
H	6.1698066	1.041694	2.5475736
H	6.0485123	-0.6599472	2.0694995
H	4.5917022	0.2188047	2.6055277
C	-3.1089045	4.9383914	-3.1532872
C	-5.2028387	5.7706365	-1.9863275
H	-5.0612548	4.0521022	-3.2657363
C	2.3345718	5.4423934	3.0243798
C	4.2921293	6.5250296	1.8269299
H	4.3925661	4.8470614	3.1636511
H	-2.435008	5.4215601	-2.4273481
H	-3.2809856	5.6481184	-3.9790048

H	-2.5871879	4.0579243	-3.5580031
H	-4.6219573	6.3073314	-1.2182672
H	-5.399761	6.4746023	-2.8115657
H	-6.1665588	5.4825095	-1.5385499
H	1.6026735	5.8075773	2.2858547
H	2.4029822	6.1919409	3.8296884
H	1.9384743	4.5096175	3.4537297
H	3.6448264	6.9435193	1.0387025
H	4.380207	7.279074	2.6263845
H	5.2901524	6.363091	1.3910786
Dy	0.1198888	-1.630495	0.002978

Dy(NHAr)NC (**3**)*

N	0.8566618	2.6768474	2.1357646
N	-1.6890778	0.6768233	-0.4968885
H	-2.3126574	-0.1219802	-0.3517756
N	1.5515066	-0.868358	0.7653568
H	2.3060288	-0.7038198	0.0924186
C	-0.2162894	-1.4918126	2.7307028
C	0.2107346	-0.4874823	3.6538141
C	-0.6166304	0.6225847	3.9070689
C	-1.8697925	0.7762179	3.2866377
C	-2.292337	-0.2524618	2.4239358
C	-1.502685	-1.3857892	2.1300955
C	1.1796194	3.5368296	2.8836741
C	-2.2061164	1.4478149	-1.5133626
C	1.7166562	-2.1057061	1.3448741
C	0.7865236	-2.5270118	2.3412581
C	1.5241351	-0.651565	4.4087068

H	-0.2584022	1.3965977	4.5823906
C	-2.7549104	2.0001013	3.4949494
H	-3.2689551	-0.1723865	1.9410439
C	-2.1367649	-2.4482216	1.2358483
C	-3.4628561	1.1345241	-2.1217935
C	-1.4654843	2.5536406	-2.0318834
C	2.8065112	-2.9775775	1.0273883
C	0.9153069	-3.7705903	2.9632717
H	2.1989748	-1.2208101	3.7518778
C	2.2188194	0.6629498	4.7776578
C	1.2706326	-1.5055912	5.666973
H	-3.7681434	1.6124489	3.7024624
C	-2.3242756	2.8779877	4.6739572
C	-2.8281669	2.82832	2.1981173
H	-3.0045195	-1.958442	0.7610015
C	-1.2570593	-2.9889401	0.1039736
C	-2.6940593	-3.5944349	2.0994313
C	-3.9657664	1.9372378	-3.1511048
C	-4.1527763	-0.1256425	-1.6978155
C	-2.0052754	3.3290591	-3.066593
C	-0.070921	2.7876287	-1.5437241
C	2.9155236	-4.2084771	1.6860351
C	3.798188	-2.4950794	0.0165212
C	1.9739394	-4.6252109	2.6350036
H	0.1909628	-4.064953	3.7269223
H	2.3550358	1.3121932	3.9037296
H	3.2080538	0.442336	5.2076075
H	1.6558451	1.232765	5.5346967
H	2.2174059	-1.6835577	6.2010864
H	0.8334359	-2.4812883	5.4113029

H	0.58108	-0.9866802	6.3531627
H	-3.0516328	3.6916695	4.8165267
H	-1.3412494	3.3392005	4.4868967
H	-2.2699176	2.3038553	5.6123521
H	-3.5458669	3.6558264	2.3059395
H	-3.1299238	2.2176639	1.3352922
H	-1.8432048	3.2680854	1.9768279
H	-1.8911481	-3.5350037	-0.6093419
H	-0.4840764	-3.6740409	0.4743855
H	-0.7612605	-2.1704348	-0.4386014
H	-1.8776702	-4.147339	2.5879309
H	-3.2536715	-4.3003629	1.4661307
H	-3.3737658	-3.2165612	2.8789912
H	-4.9215471	1.6673872	-3.6079934
C	-3.2571242	3.0479016	-3.6205586
C	-3.7936653	-1.3470319	-2.325673
C	-5.1060039	-0.1265646	-0.6511264
H	-1.4122168	4.1559096	-3.4632206
C	0.921206	1.7953701	-1.8361004
C	0.319958	3.9876455	-0.892159
H	3.7715326	-4.8490458	1.4563684
C	4.6120608	-1.3783328	0.3386956
C	3.8653966	-3.0611098	-1.2840606
H	2.0777223	-5.5926589	3.129862
H	-3.6582485	3.6645061	-4.4272266
C	-4.3802876	-2.5374551	-1.8775573
C	-2.8581062	-1.3487418	-3.5316409
C	-5.6684196	-1.3449649	-0.2374807
C	-5.5648117	1.1892928	-0.0323702
C	2.2352492	1.9885415	-1.3758799

C	0.622976	0.6975418	-2.8562344
C	1.6685154	4.164482	-0.53398
C	-0.663544	5.1297035	-0.6522204
C	5.3944862	-0.7892514	-0.6652838
C	4.7181202	-0.8789764	1.7769791
C	4.658884	-2.4319104	-2.2533278
C	3.0804543	-4.3272334	-1.629882
H	-4.1074707	-3.4813388	-2.3592642
C	-5.313391	-2.5629887	-0.8308706
H	-2.2934915	-0.4042451	-3.5041254
C	-1.8369863	-2.4931023	-3.5473595
C	-3.6935445	-1.3393267	-4.8256333
H	-6.4032227	-1.3389372	0.5687821
H	-4.7177061	1.8906564	-0.1100693
C	-6.727157	1.7868488	-0.8485225
C	-5.9682973	1.0814266	1.4444113
H	2.994019	1.230513	-1.5895865
C	2.6353805	3.1792425	-0.7422819
H	-0.4382662	0.4332336	-2.7636459
C	0.8338054	1.2925835	-4.2637692
C	1.4297343	-0.5881836	-2.6892758
H	1.9594607	5.0984822	-0.0502429
H	-1.6764876	4.7292101	-0.8156147
C	-0.4246319	6.2546601	-1.6790108
C	-0.6057701	5.7069026	0.7704597
H	6.0195753	0.0744939	-0.4197873
C	5.4079235	-1.2790066	-1.975862
H	3.8641396	-1.2931938	2.3320733
C	4.6544248	0.6456693	1.9147882
C	5.9950477	-1.4485929	2.4221481

H	4.6873253	-2.848635	-3.2621119
H	3.0388235	-4.9336885	-0.713083
C	3.7540334	-5.1890231	-2.7065344
C	1.6270495	-4.0120659	-2.0153448
C	-5.9419176	-3.8899885	-0.4225348
H	-1.2010064	-2.4750144	-2.6528603
H	-1.1826433	-2.3969264	-4.4284836
H	-2.3201137	-3.4822083	-3.6070914
H	-3.0393188	-1.2798695	-5.7109277
H	-4.3787527	-0.4787258	-4.8462891
H	-4.298802	-2.2578274	-4.9049488
H	-6.4344572	1.9525502	-1.8943443
H	-7.0432974	2.7538393	-0.4237885
H	-7.5937907	1.1054037	-0.8385582
H	-6.9137196	0.5289805	1.5692825
H	-6.1183719	2.0856805	1.8707007
H	-5.202963	0.5681257	2.0454916
C	4.1033671	3.3970909	-0.4115471
H	0.5551301	0.5548864	-5.0340921
H	1.8907404	1.5673333	-4.4150317
H	0.2167079	2.1913	-4.4112782
H	2.5165771	-0.4283162	-2.7614606
H	1.1552949	-1.2953134	-3.4853878
H	1.2197264	-1.0706446	-1.7256224
H	0.5738944	6.7001361	-1.5369238
H	-1.173758	7.0537613	-1.5570287
H	-0.4802943	5.8862936	-2.7138967
H	0.360351	6.1936634	0.9770353
H	-0.7425559	4.930936	1.5332767
H	-1.3940559	6.4666414	0.8955675

C	6.201664	-0.5642174	-3.0603403
H	4.6850584	0.9343354	2.9761679
H	3.7213741	1.0423966	1.4880002
H	5.5036907	1.142091	1.4188847
H	6.0632914	-1.1470188	3.4805258
H	6.8953442	-1.0809426	1.9022636
H	6.0015851	-2.548607	2.3742671
H	3.722856	-4.7075997	-3.6978796
H	3.2274989	-6.1523182	-2.7983164
H	4.8079182	-5.3938625	-2.4606757
H	1.5870116	-3.461951	-2.9674324
H	1.1422813	-3.3986279	-1.2445958
H	1.0454328	-4.9411004	-2.1310307
H	-5.1415001	-4.6500679	-0.4817371
C	-6.4933837	-3.9070139	1.0080229
C	-7.0346803	-4.2923879	-1.4319365
H	4.5625832	2.3970915	-0.3484102
C	4.793087	4.1424951	-1.5704927
C	4.3328586	4.1101458	0.9270482
H	6.8370602	0.1828368	-2.5530337
C	7.1292579	-1.5109677	-3.8377234
C	5.261564	0.1995749	-4.0111612
H	-6.8154391	-4.924486	1.2799382
H	-7.3717087	-3.2486686	1.1090611
H	-5.7368175	-3.5796692	1.7373579
H	-7.8556541	-3.5568173	-1.4204776
H	-7.4542283	-5.281375	-1.1844624
H	-6.6345452	-4.3329712	-2.456697
H	5.8706094	4.261517	-1.3698821
H	4.3560077	5.1461027	-1.7013844

H	4.6760163	3.5967506	-2.520169
H	5.4072044	4.1224117	1.1708104
H	3.7906356	3.6152464	1.7450529
H	3.992366	5.1575786	0.8890863
H	7.801521	-2.0551523	-3.1564487
H	7.7458101	-0.9453698	-4.554934
H	6.5540526	-2.2557503	-4.411524
H	5.8358826	0.7461783	-4.7770893
H	4.6466107	0.9279489	-3.4600916
H	4.5781756	-0.494264	-4.5278576
Dy	0.0384535	0.816102	0.9927993

Dy(NHAr)$_2$CN (**3’**)*

N	-1.7047026	0.6525358	-0.509639
H	-2.3241435	-0.1487546	-0.3616544
N	1.5679662	-0.8745386	0.7482168
H	2.3161525	-0.7165739	0.0666111
C	-0.183761	-1.4781418	2.733205
C	0.2497572	-0.4641771	3.6427348
C	-0.570213	0.655392	3.8799087
C	-1.8271231	0.8045579	3.2642539
C	-2.2599491	-0.2369741	2.4231285
C	-1.4734829	-1.3755666	2.1382339
C	-2.2378385	1.4313205	-1.5106452
C	1.7399633	-2.1047291	1.3407141
C	0.8163131	-2.5179776	2.3463564
C	1.5636619	-0.6247635	4.3974189
H	-0.2055315	1.4380001	4.541564
C	-2.7018538	2.0390992	3.4543849

H	-3.2400847	-0.1623876	1.9466686
C	-2.1161662	-2.4480503	1.261835
C	-3.5025992	1.1206192	-2.1037028
C	-1.5059716	2.5437365	-2.0278802
C	2.8314981	-2.9750821	1.0259288
C	0.9495078	-3.7582783	2.9741982
H	2.2455836	-1.177336	3.7332871
C	2.2429556	0.6907703	4.7913724
C	1.3175333	-1.4981953	5.6442166
H	-3.7134439	1.664356	3.6918346
C	-2.241779	2.9483571	4.5979845
C	-2.7937343	2.8296458	2.1353931
H	-2.989948	-1.9641423	0.7920401
C	-1.2486807	-2.998828	0.1252207
C	-2.6624656	-3.5861887	2.1429274
C	-4.0193433	1.9293529	-3.1211664
C	-4.1862987	-0.1396819	-1.6700711
C	-2.0587879	3.3233451	-3.0522941
C	-0.1066907	2.7751295	-1.550828
C	2.9452492	-4.2019585	1.6907081
C	3.8176878	-2.4897313	0.0110792
C	2.0069746	-4.6141446	2.6451157
H	0.2292984	-4.0499871	3.7427102
H	2.3634004	1.3665185	3.9351854
H	3.2379213	0.4728609	5.2093629
H	1.6770899	1.2312587	5.5677382
H	2.2657562	-1.6720022	6.1770961
H	0.8913639	-2.4754344	5.3773588
H	0.6221792	-0.9944113	6.3357526
H	-2.9575787	3.7745589	4.7263552

H	-1.2563624	3.3901091	4.3797017
H	-2.1795182	2.4034686	5.553179
H	-3.4963277	3.6710566	2.2348218
H	-3.1222006	2.199506	1.2971015
H	-1.8084262	3.2505312	1.879489
H	-1.8893591	-3.557826	-0.5721266
H	-0.4667464	-3.6745712	0.4940094
H	-0.7649757	-2.1852019	-0.4353954
H	-1.8402981	-4.1343203	2.6269377
H	-3.2293643	-4.2982036	1.5230819
H	-3.333061	-3.2010249	2.9267805
H	-4.981513	1.663005	-3.5665011
C	-3.3167069	3.0429192	-3.5931073
C	-3.8257071	-1.364195	-2.2914451
C	-5.1303446	-0.137755	-0.6152856
H	-1.4721196	4.1527545	-3.4524979
C	0.8830519	1.7861011	-1.8624746
C	0.2905547	3.9669571	-0.8864233
H	3.801344	-4.8426787	1.4618515
C	4.6280489	-1.3696466	0.3305248
C	3.8764154	-3.0509492	-1.291857
H	2.1128816	-5.5790698	3.1444304
H	-3.7278926	3.6634775	-4.3916326
C	-4.4016939	-2.5542189	-1.8290922
C	-2.8984697	-1.368119	-3.5036549
C	-5.6824499	-1.3560436	-0.1873193
C	-5.591159	1.1796319	-0.0014882
C	2.2009088	1.9734209	-1.4112981
C	0.5756014	0.6952648	-2.8868907
C	1.6435648	4.1405344	-0.5400932

C	-0.6905941	5.1058981	-0.6233887
C	5.3978356	-0.7722528	-0.6784371
C	4.7413729	-0.8726601	1.7690471
C	4.6568805	-2.4134218	-2.2660799
C	3.0946003	-4.3198225	-1.6339498
H	-4.1274883	-3.5005064	-2.3050346
C	-5.3254888	-2.5766037	-0.7738846
H	-2.3214878	-0.4311319	-3.4716389
C	-1.8927403	-2.5255331	-3.5370112
C	-3.7422124	-1.3366586	-4.7919571
H	-6.4102724	-1.3475851	0.6253037
H	-4.7541142	1.8896425	-0.1041799
C	-6.7764874	1.7546841	-0.8004925
C	-5.9621838	1.0833698	1.4846421
H	2.9570426	1.2167187	-1.6387952
C	2.608147	3.1575571	-0.7689321
H	-0.4875932	0.4395223	-2.7941826
C	0.7890197	1.2939614	-4.2922091
C	1.3714045	-0.5978092	-2.7228538
H	1.9379377	5.066826	-0.0448295
H	-1.7059072	4.6980532	-0.7522541
C	-0.4897216	6.2249444	-1.6652453
C	-0.5888036	5.699906	0.7905586
H	6.0196028	0.0946242	-0.4355571
C	5.4008634	-1.256466	-1.9910936
H	3.8987052	-1.3007357	2.3309878
C	4.6583306	0.6506812	1.913236
C	6.0322543	-1.4266363	2.4000579
H	4.6787923	-2.8260728	-3.2768622
H	3.0622032	-4.9277206	-0.7176307

C	3.7642106	-5.1767808	-2.7168959
C	1.6373041	-4.0095114	-2.0086701
C	-5.942315	-3.9038359	-0.3489679
H	-1.2543415	-2.5283522	-2.643902
H	-1.2394369	-2.4262341	-4.4185095
H	-2.3893518	-3.5070658	-3.6094676
H	-3.0930628	-1.2790649	-5.6811085
H	-4.4154826	-0.466599	-4.8012282
H	-4.3603543	-2.2462538	-4.8741192
H	-6.5072002	1.9101583	-1.8542569
H	-7.0944688	2.7234832	-0.381296
H	-7.6353303	1.0642792	-0.7646119
H	-6.8977582	0.5208709	1.6360048
H	-6.1154528	2.0904457	1.9031598
H	-5.1776345	0.5862871	2.0744851
C	4.0796659	3.3668888	-0.4492355
H	0.5048497	0.5611969	-5.0652055
H	1.8473275	1.5631167	-4.4439362
H	0.1771511	2.1971251	-4.4350407
H	2.4593946	-0.4487351	-2.8031115
H	1.0839657	-1.3045473	-3.5148262
H	1.1639595	-1.075728	-1.7560802
H	0.5124193	6.6721966	-1.5587117
H	-1.235587	7.0234426	-1.5213661
H	-0.5800587	5.8523039	-2.6957832
H	0.3665372	6.2262634	0.943816
H	-0.6555382	4.9307873	1.5694795
H	-1.3981168	6.4319685	0.9431952
C	6.181682	-0.5364538	-3.081342
H	4.6934016	0.935552	2.975461

H	3.7165726	1.0373427	1.4962228
H	5.4972912	1.1599213	1.4127723
H	6.1064214	-1.1274746	3.4586929
H	6.9223609	-1.0446306	1.8730029
H	6.0539451	-2.5263009	2.3483926
H	3.7241377	-4.6934672	-3.7069979
H	3.2411363	-6.1421173	-2.8069957
H	4.8206226	-5.3776131	-2.4787193
H	1.5888814	-3.4577289	-2.9593911
H	1.1555716	-3.3992482	-1.2334272
H	1.0583175	-4.9404629	-2.1221994
H	-5.1390396	-4.6604607	-0.4134383
C	-6.4752432	-3.9139277	1.0886984
C	-7.0458728	-4.3180207	-1.3416973
H	4.5250192	2.3629988	-0.356911
C	4.775012	4.0649868	-1.6342338
C	4.3243782	4.118696	0.8650682
H	6.7877588	0.2402714	-2.5828688
C	7.1470229	-1.4702912	-3.8284299
C	5.2289884	0.1774303	-4.0576382
H	-6.7886123	-4.9311176	1.3716141
H	-7.3555166	-3.2593129	1.1967161
H	-5.7109972	-3.5778089	1.8059641
H	-7.8701029	-3.5862098	-1.3246271
H	-7.4575677	-5.3072946	-1.0824599
H	-6.6585103	-4.3634659	-2.3711563
H	5.8547094	4.1755257	-1.4405923
H	4.3510434	5.0697467	-1.7956846
H	4.647817	3.4909527	-2.5657833
H	5.3981126	4.1093015	1.1115639

H 3.7638382 3.6695297 1.6971293

H 4.0151166 5.173803 0.7876723

H 7.8318037 -1.9738846 -3.1286768

H 7.7498995 -0.9026816 -4.5556935

H 6.6011082 -2.2487429 -4.3859933

H 5.7945286 0.7269378 -4.8279388

H 4.5848085 0.8964615 -3.5282038

H 4.5739564 -0.5475138 -4.5682698

C 0.9219266 2.7430482 2.1878883

N 1.2819671 3.6017284 2.9009634

Dy 0.0429748 0.7976036 0.9575412

Butterfly-[(NHAr)$_2$Dy(P$_4$)]$^{-}$ structure (**4**)*

Dy 0.0470843 0.0551904 -0.6697917

P 0.8879804 -0.5439666 -3.9795733

P 1.3742589 1.3130911 -2.7625445

P -1.1178236 -0.9291588 -2.9877399

N -1.8409083 0.9092036 0.372327

N 1.8703138 -0.989567 0.3023657

P -0.5547635 1.0578808 -3.9323218

C -2.3741329 1.9806524 1.0315436

C 2.3492693 -2.112843 0.9154896

C -1.5292901 3.0606608 1.451474

C -3.7813191 2.1058679 1.2889421

C 3.7428714 -2.2913047 1.2118017

C 1.4622298 -3.1921642 1.2381462

C -2.0568503 4.1324326 2.1774127

C -0.107643.0967141 0.9834761

C -4.2636601 3.2045755 2.0149268

C	-4.7728437	1.1490262	0.6948444
C	4.7619066	-1.3047075	0.7222656
C	4.1749426	-3.4512233	1.8695741
C	1.940124	-4.3270549	1.8995489
C	0.0543448	-3.1450131	0.7311827
C	-3.4185797	4.2099157	2.4904109
C	0.9220874	2.4093873	1.6829671
C	0.2171724	3.8462324	-0.1741206
C	-5.5543156	0.294787	1.5110123
C	-5.0042873	1.1609631	-0.7129041
C	5.0573144	-1.2404696	-0.6679862
C	5.4795778	-0.4714556	1.6204341
C	3.2895806	-4.465087	2.2438832
C	-0.9853279	-2.5375617	1.4845076
C	-0.2410806	-3.7245172	-0.5291603
C	2.228167	2.4367953	1.172482
C	0.6118201	1.7580959	3.0246378
C	-0.843196	4.6708109	-0.8992623
C	1.5512423	3.8841284	-0.6176872
C	-5.3379533	0.192775	3.0192271
C	-6.5768272	-0.4780094	0.9308709
C	-6.0100665	0.3459756	-1.242992
C	-4.2285443	2.1037724	-1.6326689
C	4.3355968	-2.1422247	-1.6693664
C	6.0654025	-0.3743039	-1.116426
C	5.2118673	-0.4876995	3.1235442
C	6.4824999	0.3720944	1.1206282
C	-2.2732646	-2.4729266	0.9340819
C	-0.7135191	-2.0551183	2.9034824
C	0.8326148	-4.4847402	-1.3035518

C	-1.5563541	-3.6654818	-1.0226679
C	2.5682962	3.1685066	0.0223945
C	1.5722454	0.6315813	3.4143187
C	0.5912171	2.8253752	4.1366472
C	-0.6621669	6.1640519	-0.5715328
C	-0.8732579	4.4368068	-2.4139647
C	-5.3495863	-1.2640022	3.5159619
C	-6.3872403	1.0046247	3.8011729
C	-6.8308889	-0.4629135	-0.4409741
C	-4.1595009	1.6309015	-3.0892605
C	-4.8111289	3.528283	-1.5572542
C	4.3203426	-1.5885459	-3.0991406
C	4.9288712	-3.5637085	-1.6505462
C	6.8011558	0.431453	-0.240165
C	5.2249309	0.9171597	3.7496717
C	6.2270418	-1.3798372	3.862889
C	-2.5817928	-3.0231698	-0.3196041
C	-1.6611801	-0.9496757	3.3799629
C	-0.7682489	-3.2394542	3.8883517
C	0.6567724	-5.9985618	-1.0821972
C	0.8820375	-4.1513456	-2.7984009
C	4.0116417	3.1479719	-0.4708046
C	-7.9541716	-1.2639037	-1.0930257
C	7.9513892	1.3172869	-0.7034567
C	-4.0134199	-2.9205017	-0.8308528
C	4.9463575	3.8272938	0.5475575
C	-9.0043509	-0.3201048	-1.710794
C	7.8678477	1.7310012	-2.1771073
C	-4.9253399	-3.8827889	-0.0472581
C	-4.1633878	-3.1413579	-2.3391054

C	4.1990424	3.7576738	-1.8631168
C	9.2998501	0.6325406	-0.4073617
H	2.6524303	-0.3388026	0.1495653
H	-2.5919201	0.2356357	0.1738849
H	-1.3828003	4.9417463	2.4730355
H	-3.8176	5.0577922	3.051596
H	-5.3427955	3.2870908	2.1684836
H	-7.1804265	-1.1117542	1.582596
H	-6.1752927	0.3481704	-2.3226702
H	1.7840326	4.4654093	-1.5108617
H	3.0105356	1.8712153	1.6829578
H	7.0371328	1.0109352	1.814613
H	6.2823329	-0.3366951	-2.1837964
H	5.245875	-3.5737499	2.0517123
H	3.6489638	-5.3621036	2.7530492
H	1.237503	-5.1363994	2.1179291
H	-1.7701529	-4.108837	-1.9964936
H	-3.0681519	-1.9722222	1.4912699
H	3.2867624	-2.2131712	-1.3445354
H	7.9092385	2.2387748	-0.0946411
H	4.2095836	-0.9212723	3.2699386
H	-0.4039081	1.3445068	2.9458383
H	0.3155637	-1.6672105	2.9123186
H	-4.3543682	-1.8933415	-0.6109086
H	1.8047943	-4.20252-0.8720174	
H	4.3076559	2.0842803	-0.5297396
H	-1.8223759	4.3640935	-0.5024464
H	-3.1936295	2.1461844	-1.2623345
H	-7.4957176	-1.8364479	-1.9209985
H	-4.3462648	0.6214897	3.2349371

C	-8.6371722	-2.2675913	-0.1580291
H	7.2516462	-0.9980859	3.7154325
H	6.0180754	-1.3912381	4.9460165
H	6.1962791	-2.4159509	3.4998609
H	-9.3777147	-2.8639116	-0.7149871
H	-7.914262	-2.9609927	0.2972629
H	-9.1718888	-1.7516268	0.6569563
H	-9.7835403	-0.8925724	-2.2418532
H	-9.4920614	0.2763751	-0.9218503
H	-8.5473986	0.3807744	-2.4255352
H	-5.8527866	3.5402004	-1.9212499
H	-4.7983421	3.9120277	-0.5267738
H	-4.2193552	4.2143969	-2.1854637
H	-3.4695499	2.2748048	-3.6557328
H	-3.7831925	0.5991965	-3.1638509
H	-5.1427606	1.6843397	-3.5877903
H	-7.4023064	0.6284917	3.5878543
H	-6.213274	0.9209062	4.8872856
H	-6.3578253	2.0696218	3.5332806
H	-4.6893549	-1.9046198	2.9135086
H	-5.0138124	-1.3130106	4.5643625
H	-6.361845	-1.6991195	3.4750164
H	-0.1499353	3.6094922	3.930753
H	0.3392719	2.3621353	5.1055848
H	1.5828873	3.2993459	4.2297624
H	2.5586765	1.0314188	3.688157
H	1.1870891	0.0938597	4.2948059
H	1.7087657	-0.085842.5928818	
H	4.5909296	1.6194978	3.18964
H	4.8565283	0.875055	4.7872447

H	6.2429684	1.3392884	3.7804807
H	10.1453466	1.2869035	-0.6803015
H	9.3892724	0.3772661	0.6600841
H	9.3877891	-0.3030667	-0.9841423
H	8.0154384	0.867565	-2.8462825
H	6.8908055	2.1773656	-2.4147108
H	8.6526796	2.4677417	-2.4132811
H	5.9844448	-3.5469351	-1.9717038
H	4.877935	-4.005497	-0.6449528
H	4.3702746	-4.2182153	-2.3402751
H	3.6526945	-2.1988138	-3.7263626
H	3.9482558	-0.5532356	-3.1288035
H	5.3217654	-1.6151563	-3.5625996
H	1.456311	-6.5613925	-1.5928193
H	0.6896994	-6.2499478	-0.0111237
H	-0.3131345	-6.3402713	-1.4818899
H	-0.0487183	-4.4266049	-3.3206494
H	1.0442857	-3.0768655	-2.9585198
H	1.7091604	-4.7003832	-3.2782541
H	-3.4752877	-2.4977831	-2.9095809
H	-3.9616757	-4.1906877	-2.6152742
H	-5.1956499	-2.9108785	-2.6493051
H	-4.6016575	-4.9268383	-0.1947595
H	-4.9035372	-3.6693658	1.0326586
H	-5.9679745	-3.7939573	-0.3921126
H	-0.0355515	-4.015427	3.6289048
H	-0.5527604	-2.894467	4.9138196
H	-1.773052	-3.6944978	3.8831152
H	-2.6619853	-1.351597	3.5928772
H	-1.2860056	-0.5055477	4.3154135

H	-1.7650723	-0.1552435	2.6275028
H	3.5493491	3.2642939	-2.6029126
H	3.9745354	4.8381418	-1.8611097
H	5.2453278	3.6421942	-2.1871314
H	5.9953793	3.7558357	0.2165905
H	4.6896086	4.8945913	0.6554216
H	4.8752259	3.3584175	1.5403031
H	-1.4541743	6.7652802	-1.0489057
H	-0.7043018	6.3398542	0.514509
H	0.3123934	6.5303659	-0.936521
H	0.076332	4.713181	-2.9001897
H	-1.0628823	3.3793384	-2.6443289
H	-1.674697	5.0387271	-2.8735216

Planar-[(NHAr)$_2$Dy(P$_4$)]$^-$ structure (**4**)*

Dy	-0.2239696	-0.34961	-0.7624418
N	-1.8832622	0.7598634	0.4396839
N	1.8320042	-0.9556236	0.1532543
C	-2.3551899	1.9320965	0.9631894
C	2.3674093	-2.0710485	0.7198045
C	-1.4871988	3.0354569	1.2711377
C	-3.7578278	2.1162451	1.220504
C	3.7608063	-2.2068853	1.0466146
C	1.5160637	-3.1938699	1.00549
C	-1.9970817	4.1492948	1.9493435
C	-0.046826	3.037541	0.8544541
C	-4.2211015	3.2698742	1.8656211
C	-4.7843971	1.1448879	0.7145821
C	4.7866472	-1.179114	0.6665567

C	4.2128263	-3.3689565	1.6873032
C	2.0087958	-4.3147875	1.6764505
C	0.0960132	-3.1176794	0.5370037
C	-3.3502711	4.2821651	2.2727687
C	0.9061544	2.2721854	1.5776042
C	0.3927713	3.8501544	-0.2256276
C	-5.5015351	0.3049981	1.600532
C	-5.123792	1.1450341	-0.669804
C	5.1637044	-1.0161224	-0.695048
C	5.4840101	-0.4482977	1.6665887
C	3.3570224	-4.4172861	2.0355491
C	-0.9249711	-2.6539393	1.4156761
C	-0.2420628	-3.4568234	-0.8099835
C	2.2377936	2.244088	1.1416097
C	0.5026471	1.6090582	2.889339
C	-0.5466734	4.8192555	-0.9441349
C	1.7461871	3.8150213	-0.5977696
C	-5.1419135	0.2134015	3.0817718
C	-6.5826886	-0.4544708	1.1164768
C	-6.1970981	0.3583494	-1.1032174
C	-4.363424	2.0179636	-1.6649164
C	4.4504265	-1.7775033	-1.8118582
C	6.2526184	-0.188045	-1.011106
C	5.1117192	-0.5298037	3.1459767
C	6.565876	0.3625415	1.2960993
C	-2.2244564	-2.4637997	0.9187624
C	-0.6404702	-2.4180312	2.8921856
C	0.7788713	-4.1113825	-1.7436738
C	-1.5608658	-3.2335189	-1.2586752
C	2.6781241	2.9943468	0.0444441

C	1.4065259	0.447218	3.3064243
C	0.4629059	2.6594561	4.0168783
C	-0.4627421	6.2195842	-0.3018324
C	-0.2798527	4.9593931	-2.4503632
C	-5.2119102	-1.2275054	3.61609
C	-6.037359	1.1200271	3.9461133
C	-6.9601891	-0.4299606	-0.2271603
C	-4.3962801	1.482141	-3.1009298
C	-4.8671389	3.4729206	-1.6277583
C	4.5578069	-1.0966787	-3.1817731
C	4.9485124	-3.2328542	-1.8936556
C	6.9858248	0.4907307	-0.0316871
C	5.0511859	0.8559172	3.8128323
C	6.0877784	-1.4270091	3.9298266
C	-2.5665149	-2.7269702	-0.416624
C	-1.5063275	-1.3112125	3.5062153
C	-0.8301688	-3.7272356	3.6830772
C	1.0935325	-5.5532812	-1.2933326
C	0.3412747	-4.1600901	-3.2130749
C	4.136156	2.8974341	-0.3853244
C	-8.1578758	-1.2012952	-0.7734163
C	8.2357337	1.3051921	-0.343719
C	-4.0055763	-2.515472	-0.8685795
C	5.0428175	3.6816269	0.5808294
C	-9.262588	-0.2274238	-1.2275544
C	8.3088591	1.8229893	-1.7841478
C	-4.9272408	-3.5433598	-0.1855581
C	-4.2001119	-2.546265	-2.38605
C	4.3802368	3.3277478	-1.8349001
C	9.4954124	0.48649	0.0009949

H	2.5611332	-0.2565396	-0.0168165
H	-2.6922509	0.1332012	0.3343799
H	-1.2996748	4.945382	2.2208857
H	-3.7194463	5.1695227	2.7917031
H	-5.298693.3827953	2.0141486	
H	-7.1360348	-1.0798965	1.818736
H	-6.457175	0.3600003	-2.1644293
H	2.0688695	4.4241446	-1.4425317
H	2.9606336	1.6152767	1.6654376
H	7.1071865	0.9140883	2.0711604
H	6.5382412	-0.083426	-2.0574929
H	5.2831684	-3.4567286	1.8912113
H	3.7370544	-5.3079051	2.5403834
H	1.3257835	-5.1411751	1.8916386
H	-1.8078085	-3.4603392	-2.2935075
H	-2.9960767	-2.0743416	1.584854
H	3.3779897	-1.8057903	-1.5618797
H	8.2201783	2.1838125	0.3269137
H	4.1101289	-0.9839282	3.206136
H	-0.5199756	1.2272944	2.7539999
H	0.4181559	-2.1307201	2.9716445
H	-4.3106555	-1.5163114	-0.5109626
H	1.7045409	-3.5154717	-1.6747983
H	4.4175072	1.8326267	-0.3047453
H	-1.5726529	4.4389316	-0.8141539
H	-3.3056614	2.0252594	-1.3567678
H	-7.8047747	-1.7420092	-1.6717201
H	-4.1036813	0.5701988	3.1827277
C	-8.7355065	-2.2393738	0.1949193
H	7.1133049	-1.0243255	3.8708574

H	5.8008692	-1.4753547	4.9939182
H	6.1037407	-2.4522441	3.5354637
H	-9.5374634	-2.8139393	-0.2959462
H	-7.9685723	-2.9495489	0.5399377
H	-9.1721803	-1.7547833	1.0840445
H	-10.1040749	-0.7722414	-1.6881058
H	-9.6493284	0.3392311	-0.3643093
H	-8.8808963	0.4993091	-1.960507
H	-5.9230816	3.5275336	-1.94487
H	-4.7836163	3.9009088	-0.6186568
H	-4.267343	4.0943113	-2.3129237
H	-3.7060108	2.0681859	-3.7262397
H	-4.0731845	0.4309569	-3.1473659
H	-5.4018111	1.5568481	-3.5505715
H	-7.0970736	0.834317	3.8329859
H	-5.7694019	1.0272091	5.0122089
H	-5.9344323	2.1751838	3.6606624
H	-4.6696278	-1.9306277	2.9662245
H	-4.7740207	-1.2827317	4.6255046
H	-6.252832	-1.5830475	3.6917219
H	-0.233378	3.4763751	3.7828204
H	0.1390011	2.1939787	4.96353
H	1.466257	3.0909645	4.1717222
H	2.4049931	0.8073593	3.5899759
H	0.9891996	-0.0623001	4.1881272
H	1.5254244	-0.2804366	2.4922502
H	4.4321702	1.5584994	3.2369127
H	4.6248866	0.7732896	4.8256791
H	6.0545284	1.3016507	3.913883
H	10.4110023	1.0816984	-0.1573261

H	9.4761077	0.1515591	1.049772
H	9.5531187	-0.41141-0.6363468	
H	8.4178406	0.9956784	-2.5044044
H	7.4046423	2.3883764	-2.0541407
H	9.1806628	2.4854119	-1.9090996
H	6.0189096	-3.2622064	-2.1609963
H	4.815284	-3.7568482	-0.9359863
H	4.3859517	-3.7828315	-2.6665402
H	3.8947763	-1.6046284	-3.8976439
H	4.2437032	-0.0421094	-3.1315297
H	5.5844961	-1.1366256	-3.5855604
H	1.812094	-6.0107911	-1.9929931
H	1.5332795	-5.5883821	-0.2901146
H	0.175594	-6.1651062	-1.2988346
H	-0.4829861	-4.8795799	-3.3585029
H	0.0188221	-3.1798631	-3.5911927
H	1.1865398	-4.4918356	-3.8350846
H	-3.4972905	-1.8719707	-2.8993868
H	-4.0535182	-3.5629208	-2.7895616
H	-5.2266873	-2.2332574	-2.6324317
H	-4.641251	-4.5695315	-0.4713285
H	-4.8821509	-3.4672174	0.9116242
H	-5.9707632	-3.3760492	-0.4930611
H	-0.168804	-4.5236252	3.3159197
H	-0.6085803	-3.5628391	4.7506499
H	-1.8723931	-4.0787399	3.5990987
H	-2.5376126	-1.6568775	3.6663507
H	-1.108297	-1.0168743	4.489245
H	-1.5427868	-0.4239282	2.857805
H	3.6946043	2.8043039	-2.5185142

H	4.238873	4.4144265	-1.9646326
H	5.4153046	3.0931543	-2.128986
H	6.1034601	3.5594945	0.3052422
H	4.7983512	4.7570885	0.5534554
H	4.9199086	3.3314659	1.6170836
H	-1.1642846	6.9135207	-0.795389
H	-0.7004339	6.2009705	0.7698788
H	0.5567757	6.626238	-0.4141101
H	0.6781966	5.4683293	-2.6496064
H	-0.2656554	3.9833719	-2.9555978
H	-1.075424	5.5676548	-2.9118757
P	1.263398	1.2224117	-2.7671485
P	-1.1160012	-0.5308845	-3.6141838
P	1.0317026	-0.8272234	-3.4329456
P	-0.8833379	1.5251998	-2.9641322

References

1 Benner, F.; Jena, R.; Odom, A. L.; Demir, S., Magnetic Hysteresis in a Dysprosium Bis(amide) Complex *J. Am. Chem. Soc.* **2025**, *147*, 8156-8167.

2 Chen, X.; Li, Q.; Gong, Y.; Andrews, L.; Liebov, B. K.; Fang, Z.; Dixon, D. A. *Inorg. Chem.*, **2017**, *56*, 5060–5068.

3 Tarlton, M. L.; Yu, X.; Ward, R. J.; Kelley, S. P.; Autschbach, J.; Walensky, J. R. *Chem. – Eur. J.*, **2021**, *27*, 14396–14400.

4 Bouzidi, Y.; Belkhiri, L.; Ephritikhine, M.; Halet, J.-F.; Boucekkine, A. *J. Organomet. Chem.*, **2017**, *847*, 82–89.

5 Jena, R.; Benner, F.; Delano, F.; Holmes, D.; McCracken, J.; Demir, S.; Odom, A. L. *Chem. Sci.*, **2023**, *14*, 4257–4264.

6 Bain, G. A.; Berry, J. F. *J. Chem. Educ.*, **2008**, *85*, 532.

7 Kollmar, C.; Sivalingam, K.; Helmich-Paris, B.; Angeli, C.; Neese, F. *J. Comput. Chem.*, **2019**, *40*, 1463–1470.

8 Neese, F. *WIREs Comput. Mol. Sci.*, **2012**, *2*, 73–78.

9 Neese, F. *WIREs Comput. Mol. Sci.*, **2022**, *12*, e1606.

10 Douglas M.; Kroll, N. M. *Ann. Phys.*, **1974**, *82*, 89–155.

11 Hess, B. A. *Phys. Rev. A*, **1985**, *32*, 756–763.

12 Weigend, F. Ahlrichs, R. *Phys. Chem. Chem. Phys.*, **2005**, *7*, 3297.

13 Rolfes, J. D.; Neese, F.; Pantazis, D. A. *J. Comput. Chem.*, **2020**, *41*, 1842–1849.

14 Aravena, D.; Neese, F.; Pantazis, D. A. *J. Chem. Theory Comput.*, **2016**, *12*, 1148–1156.

15 Stoychev, G. L.; Auer, A. A.; Neese, F. *J. Chem. Theory Comput.*, **2017**, *13*, 554–562.

16 Staroverov, V. N.; Scuseria, G. E.; Tao J.; Perdew, J. P. *J. Chem. Phys.*, **2003**, *119*, 12129–12137.

17 Staroverov, V. N.; Scuseria, G. E.; Tao J.; Perdew, J. P. *J. Chem. Phys.*, **2004**, *121*, 11507–11507.

18 Sheldrick, G. M. *Acta Crystallogr. Sect. C Struct. Chem.*, **2015**, *71*, 3–8.

19 Dolomanov, O. V.; Bourhis, L. J.; Gildea, R. J.; Howard J. A. K.; Puschmann, H. *J. Appl. Crystallogr.*, **2009**, *42*, 339–341.

20 Gaussian 16, Revision C.01, M. J. Frisch, G. W. Trucks, H. B. Schlegel, G. E. Scuseria, M. A. Robb, J. R. Cheeseman, G. Scalmani, V. Barone, G. A. Petersson, H. Nakatsuji, X. Li, M. Caricato, A. V. Marenich, J. Bloino, B. G. Janesko, R. Gomperts, B. Mennucci, H. P. Hratchian, J. V. Ortiz, A. F. Izmaylov, J. L. Sonnenberg, D. Williams-Young, F. Ding, F. Lipparini, F. Egidi, J. Goings, B. Peng, A. Petrone, T. Henderson, D. Ranasinghe, V. G. Zakrzewski, J. Gao, N. Rega, G. Zheng, W. Liang, M. Hada, M. Ehara, K. Toyota, R. Fukuda, J. Hasegawa, M. Ishida, T. Nakajima, Y. Honda, O. Kitao, H. Nakai, T. Vreven, K. Throssell, J. A. Montgomery, Jr., J. E. Peralta, F. Ogliaro, M. J. Bearpark, J. J. Heyd, E. N. Brothers, K. N. Kudin, V. N. Staroverov, T. A. Keith, R. Kobayashi, J. Normand, K. Raghavachari, A. P. Rendell, J. C. Burant, S. S. Iyengar, J. Tomasi, M. Cossi, J. M. Millam, M. Klene, C. Adamo, R. Cammi, J. W. Ochterski, R. L. Martin, K. Morokuma, O. Farkas, J. B. Foresman, and D. J. Fox, Gaussian, Inc., Wallingford CT, 2019.Sonnenberg, D. Williams-Young, F. Ding, F. Lipparini, F. Egidi, J. Goings, B. Peng, A. Petrone, T. Henderson, D. Ranasinghe, V. G. Zakrzewski, J. Gao, N. Rega, G. Zheng, W. Liang, M. Hada, M. Ehara, K. Toyota, R. Fukuda, J. Hasegawa, M. Ishida, T. Nakajima, Y. Honda, O. Kitao, H. Nakai, T. Vreven, K. Throssell, J. A. Montgomery, Jr., J. E. Peralta, F. Ogliaro, M. J. Bearpark, J. J. Heyd, E. N.

Brothers, K. N. Kudin, V. N. Staroverov, T. A. Keith, R. Kobayashi, J. Normand, K. Raghavachari, A. P. Rendell, J. C. Burant, S. S. Iyengar, J. Tomasi, M. Cossi, J. M. Millam, M. Klene, C. Adamo, R. Cammi, J. W. Ochterski, R. L. Martin, K. Morokuma, O. Farkas, J. B. Foresman, and D. J. Fox, Gaussian, Inc., Wallingford CT, 2019.

21 M. J. Frisch, G. W. Trucks, H. B. Schlegel, G. E. Scuseria, M. A. Robb, J. R. Cheeseman, G.Scalmani, V. Barone, G. A. Petersson, H. Nakatsuji, X. Li, M. Caricato, A. V. Marenich, J. Bloino, B. G. Janesko, R. Gomperts, B. Mennucci, H. P. Hratchian, J. V. Ortiz, A. F. Izmaylov, J. L. Sonnenberg, D. Williams Young, F. Ding, F.Lipparini, F. Egidi, J. Goings, B. Peng, A. Petrone, T .Henderson, D. Ranasinghe, V. G. Zakrzewski, J. Gao, N. Rega, G. Zheng, W .Liang, M. Hada, M. Ehara, K. Toyota, R. Fukuda, J. Hasegawa, M. Ishida, T. Nakajima, Y. Honda, O. Kitao, H. Nakai, T. Vreven, K. Throssell, J. A. Montgomery, Jr, J. E. Peralta, F. Ogliaro, M. J. Bearpark, J. J. Heyd, E. N. Brothers, K. N. Kudin, V. N. Staroverov, T. A. Keith, R. Kobayashi, J. Normand, K. Raghavachari, A. P. Rendell, J. C. Burant, S. S. Iyengar, J. Tomasi, M. Cossi, J. M. Millam, M. Klene, C. Adamo, R. Cammi, J. W. Ochterski, R. L. Martin, K. Morokuma, O. Farkas, J. B. Foresman and D. J. Fox, Gaussian program suite (revision B01), 2016.

22 Humphrey, W.; Dalke, A.; Schulten, K. *J. Mol. Graph.*, **1996**, *14*, 33–38.

23 Dolg, M.; Stoll, H.; Savin A.; Preuss, H. *Theor. Chim. Acta*, **1989**, *75*, 173–194.

24 Dolg, M.; Stoll, H.; Savin A.; Preuss, H. *Theor. Chim. Acta*, **1993**, *85*, 441–450.

25 Smith, D. G. A.; Burns, L. A.; Patkowski K.; Sherrill, C. D. *J. Phys. Chem. Lett.*, **2016**, *7*, 2197–2203.

26 Grimme, S.; Antony, J.; Ehrlich S.; Krieg, H. *J. Chem. Phys.*, **2010**, *132*, 154104.
